# Snow-Dependent Biogeochemical Cycling of Polycyclic Aromatic Hydrocarbons at Coastal Antarctica

**DOI:** 10.1021/acs.est.2c05583

**Published:** 2023-01-19

**Authors:** Jon Iriarte, Jordi Dachs, Gemma Casas, Alicia Martínez-Varela, Naiara Berrojalbiz, Maria Vila-Costa

**Affiliations:** Department of Environmental Chemistry, Institute of Environmental Assessment and Water Research, IDAEA-CSIC, 08034Barcelona, Catalunya, Spain

**Keywords:** polycyclic aromatic hydrocarbons, PAH, coastal Antarctica, biodegradation, biogeochemical processes, marine bacterial communities

## Abstract

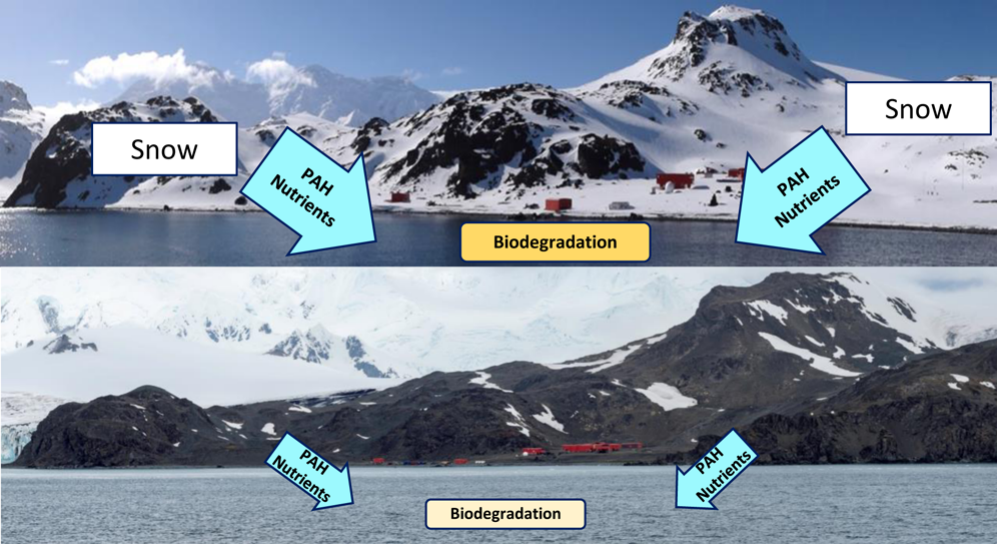

The temporal trend of polycyclic aromatic hydrocarbons (PAHs) in coastal waters with highly dynamic sources and sinks is largely unknown, especially for polar regions. Here, we show the concurrent measurements of 73 individual PAHs and environmental data, including the composition of the bacterial community, during three austral summers at coastal Livingston (2015 and 2018) and Deception (2017) islands (Antarctica). The Livingston 2015 campaign was characterized by a larger snow melting input of PAHs and nutrients. The assessment of PAH diagnostic ratios, such as parent to alkyl-PAHs or LMW to HMW PAHs, showed that there was a larger biodegradation during the Livingston 2015 campaign than in the Deception 2017 and Livingston 2018 campaigns. The biogeochemical cycling, including microbial degradation, was thus yearly dependent on snow-derived inputs of matter, including PAHs, consistent with the microbial community significantly different between the different campaigns. The bivariate correlations between bacterial taxa and PAH concentrations showed that a decrease in PAH concentrations was concurrent with the higher abundance of some bacterial taxa, specifically the order *Pseudomonadales* in the class *Gammaproteobacteria*, known facultative hydrocarbonoclastic bacteria previously reported in degradation studies of oil spills. The work shows the potential for elucidation of biogeochemical processes by intensive field-derived time series, even in the harsh and highly variable Antarctic environment.

## Abstract 2

The concurrent measurement of PAHs and environmental variables, including the microbial composition, shows a large yearly variability of cycling at coastal Antarctica, especially microbial degradation, linked to the role of snow melting as a biogeochemical driver.

## Introduction

Polycyclic aromatic hydrocarbons (PAHs) are ubiquitous in the oceans and polar regions.^1−4^ Even though most emissions of PAHs are land-based due to the use of fossil fuels and biomass burning,^5^ PAHs can reach remote regions, such as Antarctica, through long-range atmospheric transport and deposition.^1,6^ Diffusive air–water exchange is the main input of PAHs for most oceanic regions.^3,4,7^ However, for maritime Antarctica, snow scavenging of atmospheric PAHs and the subsequent melting of the snowpack play a key role as a pollutant input to coastal seawater.^6^ Indeed, PAH concentrations in seawater have been shown to increase gradually during the austral summer due to the melting of the snowpack accumulated during the preceding winter–spring, which, when melted, flashed-out pollutants from land to the sea.^6^ Nevertheless, this was described for the 2014–2015 austral summer, after an important snowpack was accumulated and melted. The extent of the snow events and snowpack is highly variable yearly, and thus there is a dearth of understanding of the variability of PAH cycling depending on snow accumulation, which in turn depends on the weather patterns in the Antarctic Peninsula region.^8,9^ The understanding of the sources and dynamics of PAHs in coastal areas, especially in polar regions, is especially important for PAHs. PAHs and other semivolatile aromatic-like compounds are among the main families of anthropogenic organic chemicals entering the ocean and account for a relevant fraction of the anthropogenic dissolved organic carbon in the marine environment.^10,11^ Furthermore, together with other families of pollutants, they form complex mixtures of organic pollutants that are toxic to marine phytoplankton.^12^

Once in the water column, the fate of PAHs is dominated by the biological pump and degradation.^11^ The biological pump is the settling of organic matter-bound PAHs into deep waters and sediments. This is the sinking of PAHs that have been incorporated in phytoplankton or partitioned to detritus and other organic carbon pools driven by PAH hydrophobicity. However, the biological pump removes only 1% of the atmospheric inputs of PAHs.^11^ The vast majority of PAHs entering the ocean are degraded in the photic zone. Even though photodegradation can occur, the overall PAH sink is dominated by microbial degradation.^11,13^ Despite this relevance, the study of microbial degradation of PAHs under field conditions has been mainly centered under scenarios of high concentrations, such as those found in oil spills,^14,15^ with few field studies addressing the degradation of PAHs when these occur at their background levels.^16^

Hydrocarbon-degrading bacteria are known as hydrocarbonoclastic bacteria (HCB), which include obligate HCB, that is, bacteria that only grow using hydrocarbons as carbon and energy sources, and facultative HCB, those able to grow with alternative carbon sources.^17^ HCB are naturally found at low abundances but can become predominant shortly after pulses of hydrocarbons (both aliphatic and aromatic).^18−20^ HCB are also ubiquitous in remote environments such as polar sea ice, the surface microlayer, or deep marine nonpolluted seawater.^21−25^ HCB belong to different phylogenetic groups, including members in the most common marine dominant classes, *Gammaproteobacteria*, *Alphaproteobacteria*, and *Flavobacteria*. In polar environments, there is a suite of microbial responses, including biodegradation, due to exposure to mixtures of anthropogenic dissolved organic carbon (ADOC).^16,26^ Other works assessing the influence of ADOC on polar bacteria at ultratrace concentrations have reported the growth of the rare biosphere, in some cases, HCB.^26,27^ Nevertheless, studies attempting to compare HCB presence to the occurrence of PAH in seawater and assess their mutual interactions are still scarce at background environmental levels, despite the reported HCB and PAH co-occurrence under scenarios of oil spills.^16,28,29^ In addition, the interactions of HCB and PAHs have never been reported for time series of measurements. Dynamics of PAHs over the austral summer may depend on the highly variable snow deposition events, glacier melting, and other environmental conditions. Characterizing potential consumers might provide hints toward a better elucidation of the biogeochemistry of PAHs, and especially biodegradation, in these ecosystems.

The objectives of this work were (i) to contribute with the largest data set to date on PAHs in Antarctic seawater and plankton as well as HCB communities, comprising three-time series of measurements in maritime Antarctica during three austral summers, (ii) to explore the influence of environmental factors, and especially microbial communities including HCB, on the occurrence and variability of PAHs in the water column under scenarios of variable strength of snow melting, (iii) explore the potential of *in situ* field biogeochemical assessments to identify the role of HCB in the PAH degradation in the marine environment.

## Materials and Methods

### Site Description

This study comprises samples collected during three different sampling campaigns performed during the austral summer. Two of the campaigns took place at South and False Bays of Livingston Island (62° 39′ S, 60° 23′ W) from December 1st, 2014, to March 1st, 2015, and from January 8th to March 1st, 2018, respectively. The third campaign took place at the inner bay of Deception Island (62° 59′ S, 60° 37′ W) from January 22nd to February 20th, 2017. Both islands are part of the South Shetland Archipelago, lying 150 km north of the Antarctic Peninsula. Livingston Island has a surface area of 850 km^2^ and it is mostly covered by glaciers. The only two research stations on the island are located at South Bay: the Spanish Antarctic research station Juan Carlos I and the Bulgarian research station Saint Kliment Ohridski. Both stations operate only during the summer months, accounting for a maximum population of 50 people. Deception Island, which has a surface area of 72 km^2^, is a volcanic island where over half of the area is covered by glaciers, with Port Foster in the caldera. Currently, there are two research stations that are only operative during the summer months: the Spanish Antarctic research station Gabriel de Castilla and the Argentinian Antarctic station Deception. The conditions at coastal Livingston are representative of the maritime sector of the Antarctic Peninsula and other Antarctic sectors receiving important snow inputs and under the influence of glaciers. In contrast, Port Foster at Deception is a particular site that may not be fully representative of other Antarctic coastal sites due to the geometry and influence of the volcano.

### Sampling

**Figure 1:**
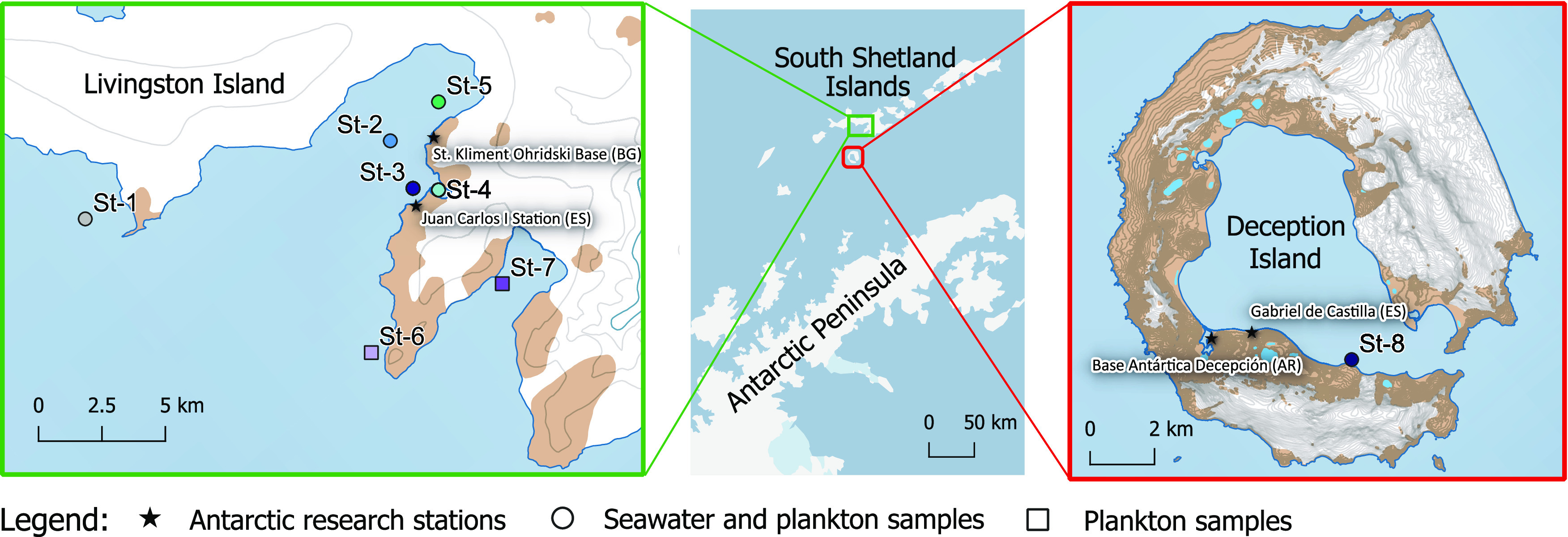
Location of the sampling sites for water and plankton samples at coastal Livingston (left panel) and Deception Islands (right panel) in the South Shetlands Islands (Antarctica). Figure created using Quantarctica version 3.2.^30^

Simultaneous plankton and seawater samples were taken from a rigid inflatable boat at 8 different locations (Figure 1, Table S1).^30^ Station 1 (St-1) was located off-shore Punta Hanna at the eastern side of South Bay. Stations 2–5 (St-2–St-5) were located at different locations in South Bay and at different distances from the Pimpirev and Johnsons glaciers. In fact, St-4 is located in a small bay only 200 m from the Johnsons glacier. All five stations for Livingston Island were sampled during the 2018 campaign (Livingston 2018), while only St-3 and St-4 were sampled during the 2014–2015 campaign (Livingston 2015). Additional plankton samples were taken at stations 6 and 7, next to Miers Bluff and inside False Bay, respectively. Conversely, Station 8 (St-8) is located inside Port Foster Bay at Deception Island and was sampled during the 2016–2017 campaign (Deception 2017).

Concurrently with water sampling, CTD depth profiles were taken to evaluate water temperature, salinity, turbidity, fluorescence, and photosynthetic active radiation (PAR) (Table S2).

In total, 46 surface seawater samples were collected. Twenty-six, seven, and thirteen during the Livingston 2015, Deception 2017, and Livingston 2018 sampling campaigns, respectively. Up to 100–120 L of seawater at 0.5–1 m depth was collected in 20 L aluminum jerry cans and transported to the research stations for their immediate filtration and extraction. This took place outside of the research stations to keep the temperature at ambient levels (1–4 °C), so no repartitioning between dissolved and particle phases occurred during sample manipulation. This also avoided any potential contamination from indoor air. Briefly, seawater was filtered through a precombusted and preweighed glass fiber filter (140 mm, GF/F Whatman) and then through a precleaned XAD-2 adsorbent (50 g, Supelco), packed in stainless steel columns. XAD-2 columns were stored at 4 °C for refrigerated transport until further analysis in a clean lab in Barcelona. GF/F filters were wrapped in precombusted aluminum foil and stored at −20 °C in air-tight plastic bags. The GF/F filters corresponding to surface particles were not analyzed for this study.

Fifty plankton samples were collected using a conical plankton net with a 50 μm mesh size by three vertical hauls from the bottom to the surface. The sampling depths ranged from 12 m to 60 m (Table S1). The samples were collected at 8 different sampling stations (St-1–St-8) (Figure 1 and Table S1). Samples were filtered through precombusted and preweighed glass fiber filters (47 mm, GF/D Whatman). The filters containing plankton samples were wrapped in precombusted aluminum foil and stored at −20 °C in air-tight plastic bags until further analysis in a clean lab in Barcelona.

Fifteen milliliters of the water samples was taken and kept at −20 °C for analyzing dissolved inorganic nutrients: nitrate + nitrite (NO_3_^–^ + NO_2_^–^), ammonium (NH_4_^+^), and phosphate (PO_4_^3–^). For the absolute quantification of bacterial abundance (BA) by flow cytometry, 1.8 mL of the water sample was fixed with a 1% buffered paraformaldehyde solution (pH 7.0) plus 0.05% glutaraldehyde (P + G), left at room temperature in the dark for 10 min, and frozen and stored at −80 °C until further processing.

Samples for 16S rDNA library construction were collected from surface water and analyzed as reported elsewhere.^24^ Four liters of each sample was prefiltered through 3 μm pore size 47 mm diameter polytetrafluoroethylene filters (Millipore, Billerica, MA) to remove grazers and the particle-attached living fraction and sequentially onto 0.2 μm pore size 47 mm PTFE (Millipore, Billerica, MA) filters to capture the free-living bacteria fraction, using a peristaltic pump with a flow of <50 mL min^–1^. Each filter was placed in 1 mL of the lysis buffer (50 mM Tris HCl, 40 mM EDTA, 0.75 M sucrose). All filters were stored at −20 °C until further processing.

### Analytical Procedures for PAH Determination

The procedures followed for the extraction, identification, and quantification of PAHs are described in Annex S1 in the Supporting Information and were the same for the three campaigns. Quality assurance and quality control are reported in Annex S2 in the Supporting Information. Briefly, strict measures were taken to clean the material used during sampling and analysis. Field blanks consisted of GF/D filters and XAD-2 columns that followed the same process as the samples, albeit without the pass of plankton or water. Recoveries and limits of quantification are summarized in Tables S3 and S4 in the Supporting Information. Recoveries ranged between 41 and 100% and between 19 and 100% for the plankton and water-dissolved phases, respectively. Concentrations were corrected for surrogate recoveries to account for the different recoveries of the lighter surrogates for the 2017–2018 water sampling.

The following parent and alkylated PAHs were analyzed: phenanthrene (Phe), anthracene (Ant), dibenzonthiophene (DBT), fluoranthrene (Flt), pyrene (Pyr), benzo[*a*]anthracene (B[*a*]ant), chrysene (Cry), benzo[*b*]fluoranthene (B[*b*]f), benzo[*k*]fluoranthene (B[*k*]f), benzo[*e*]pyrene (B[*e*]pyr), benzo[*a*]pyrene (B[*a*]pyr), perylene (Pery), dibenzo[*a*,*h*]anthracene (Dib[*a*,*h*]ant), benzo[*g*,*h*,*i*]perylene (B[*g*,*h*,*i*]pery), indeno[1,2,3-cd]pyrene (In[1,2,3-cd]pyr), benzo[ghi]fluoranthrene (B[*g*,*h*,*i*]f), methylfluorene (∑MFlu, sum of 4 isomers), methylphenanthrenes (∑MPhe, sum of 5 isomers), dimethylphenanthrenes (∑DMPhe, sum of 10 isomers), trymethylphenanthrenes (∑TMPhe, sum of 12 isomers), methyldibenzonthiophenes (∑MDBT, sum of 3 isomers), dimethyldibenzonthiophenes (∑DMDBT, sum of 6 isomers), methylpyrenes (∑MPyr, sum of 5 isomers), dimethylpyrenes (∑DMPyr, sum of 8 isomers), and methylchrysenes (∑MCry, sum of 4 isomers).

PAH concentrations in the water-dissolved and plankton phases for the Livingston 2015 campaign have been reported in a companion publication and are used here when needed.^6^

### Bacterial Abundance and Nutrient Quantification

Bacterial abundance was estimated by flow cytometry as described elsewhere.^31^ Dissolved inorganic nutrients were analyzed by standard segmented flow with colorimetric detection using a SEAL Analyzer AA3 HR.^32^ Detection limits (defined as three times the standard deviation of 10 replicates at 50% diluted samples) were 0.006 μM for NO_3_^–^, 0.003 μM for NO_2_^–^, 0.003 μM for NH_4_^+^, and 0.01 μM for PO_4_^3–^ (Table S5).

### Nucleic Acids Extraction and Sequencing

After unthawing, samples were incubated with lysozyme, proteinase K, and sodium dodecyl sulfate (SDS), and nucleic acids were extracted simultaneously with phenol/chloroform/isoamyl alcohol (25:24:1) and chloroform/isoamyl alcohol (24:1).^33^ The resulting solution was concentrated to 200 μL using an Amicon Ultra 10-kDa filter unit (Millipore). Partial bacterial 16S gene fragments of both DNA were amplified using primers 515F-Y and 926R^34^ plus adapters for Illumina MiSeq sequencing. The PCR reaction mixture was thermocycled at 95 °C for 3 min, 30 cycles at 95 °C for 45 s, 50 °C for 45 s, and 68 °C for 90 s, followed by a final extension of 5 min at 68 °C. PCR amplicon sizes were checked in tris-acetate-EDTA (TAE) agarose gels. Illumina MiSeq sequencing was conducted at the Pompeu Fabra University Sequencing Service. The complete nucleotide sequence data set generated and analyzed in this study was deposited in the sequence read archive (SRA) under the bioproject accession # PRJNA739708 and SUB9892364.

### Bioinformatics

DADA2 v1.4 was used to differentiate the 16S V4-5 amplicon sequence variants (ASVs) and remove chimeras (parameters: maxN = 0, maxEE = 2,4, trunclen = 227,210).^35^ DADA2 resolves ASVs by modeling the errors in Illumina-sequenced amplicon reads. The approach is threshold-free, inferring exact variants up to 1 nucleotide difference using the quality score distribution in a probability model. Previously, spurious sequences and primers were trimmed using cutadapt v.1.16.^36^ Taxonomic assignment of the ASVs was performed with the SILVA algorithm classifier against SILVA database release 138 (Quast et al., 2013). Taxonomic assignment of the ASVs was performed with the RDP algorithm classifier against RDP database release 11.5.^37^ ASVs classified as Mitochondria or Chloroplast were removed. The final ASV table contained 69 samples, obtaining for the entire sample set 7359 ASVs from 16S rRNA gene V4-5 fragments, from which 1844 were unique. The maximum and minimum number of unique ASVs per sample was 425 and 49, respectively, with an average of 153.3 ± 76.4. To allow for comparisons between samples, samples below 5000 reads/sample were filtered, and rarefaction was done to the minimum sequencing depth (6131 reads/sample) with the rrarefy() function from package vegan v2.5-7 in the R environment.^38^ The identification of HCB was performed following the same approach reported elsewhere.^16^ The list of HCB genera includes genera either collected from hydrocarbon-polluted environments, usually oil spills, observed to have stimulated growth following hydrocarbon exposure (both aromatic and aliphatic hydrocarbons), or showing hydrocarbon catabolic activity, both from isolates and from marine environments, after metagenome-assembled genomes reconstruction.

### Validation of 16S Sequencing Data by Quantitative PCR (qPCR)

The relative abundances assessed by 16S sequencing were validated using qPCR to estimate the abundances of 16S rRNA of one group of HCB, the genera Colwellia, psychrophilic genera of obligate HCB.^25^ Quantitative PCR was performed using a LightCycler 480 SYBR Green I Master (A F. Hoffmann–La Roche AG, Inc) in a LightCycler 480 II (A F. Hoffmann–La Roche AG, Inc), in 20μL reaction volumes on 96-well plates. PCR conditions were reproduced using the Colwellia-specific primers pair COL134 and COL209^39^ following Krolicka et al.^40^ All samples were run as technical duplicates along with the quantification curve to reduce variability between assays. The plasmid used for the quantification curves was pNORM conjugative plasmid.^41^ Quantification limits (LOQ) were established as the minimum amount of plasmid that could be detected without interference from the negative control. The quality criteria within the standard curve were *R*^2^ > 0.99, and a slope between −3.1 and −3.4. The accepted efficiency of the reactions ranged from 97 to 100%. Melting curves were obtained to confirm amplification specificity. The amplification protocol was performed following the manufacturer’s guidelines.

### Statistical Analysis

All statistical analyses were carried out with R v4.1.0 software (http://www.r-project.org/). Analysis of variance (ANOVA) followed by a post-hoc Tukey HSD was used to test significant differences among samples, using the stast v4.1.0 and agricolae v1.3.5 packages in R. Package ggpubr v0.4.0 was used for Pearson correlations (*p* ≤ 0.05). A nonmetric multidimensional scaling (NMDS) approach was used to assess the clustering of the samples based on their ASV composition using function metaMDS from vegan v2.5-7 package in R.^38^ PERmutational Multivariate ANOVA^42^ was used with function Adonis from package vegan to elucidate the factors (i.e., campaign, site, date) that significantly structured the microbial communities. Heatmaps (heatmap.2 function from the gplots v3.1.1 package) were used to visualize Pearson correlations between bacterial taxa and environmental data among the different samples, whereas PLS2 (plsdepot v0.1.17 package) was used to explore the correlation between the relative bacterial taxa abundance (predictive, X-matrix) and the environmental data in each sample. Further graphs were plotted using package ggplot v3.3.5, also in the R environment.^43^

## Results and Discussion

### Occurrence of PAHs in Seawater and Plankton

**Figure 2:**
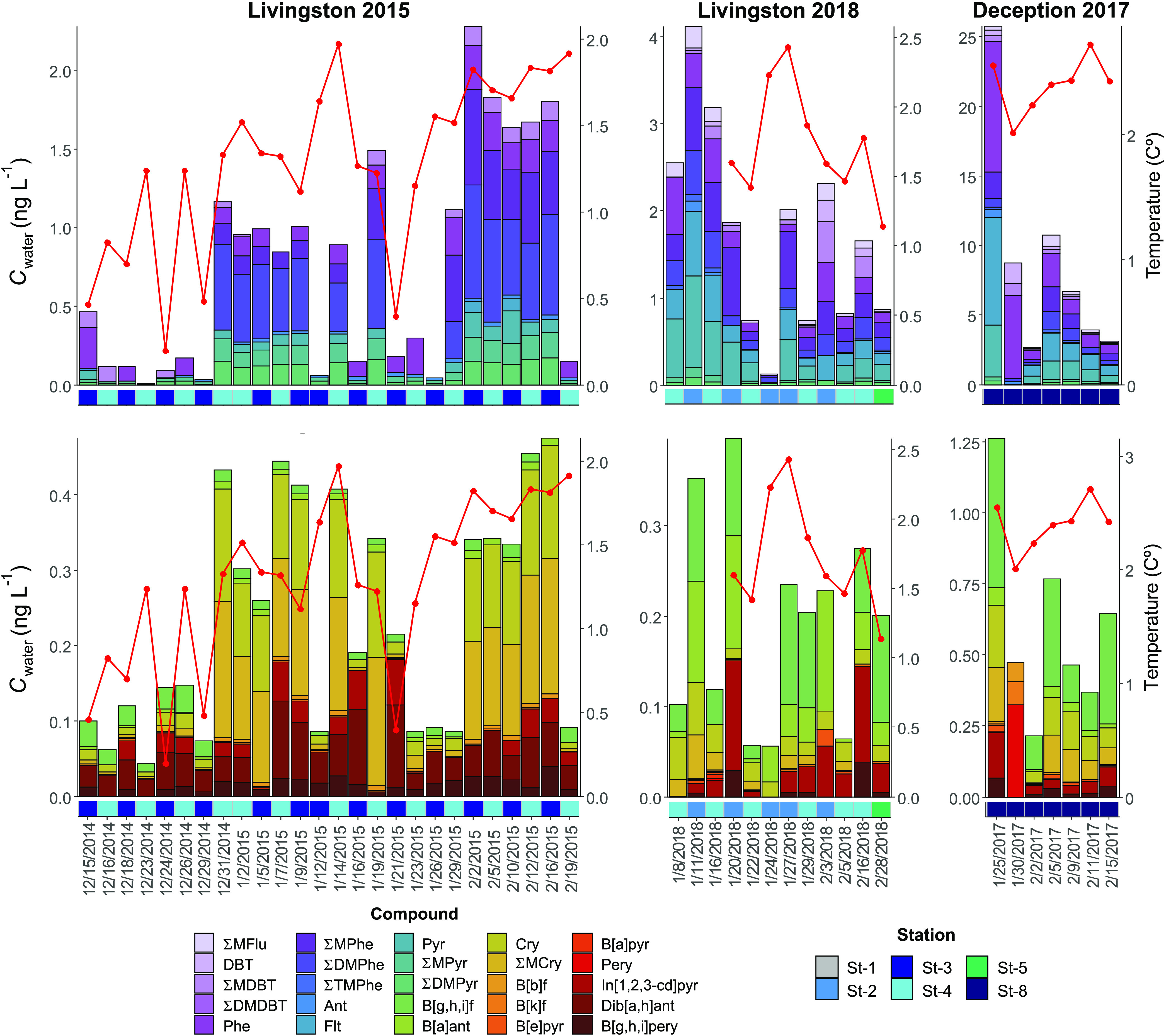
Concentrations of low MW PAHs (upper panels) and high MW PAHs (lower panels) in the water-dissolved phase for the three sampling campaigns at Livingston and Deception Islands. The red line indicates the seawater temperature.

The concentrations of the 73 targeted parent and alkylated PAHs in the water-dissolved phase and plankton phases at coastal Livingston and Deception Islands are reported in Tables S6 and S7 and shown in Figures 2 and S2. Dissolved-phase concentrations of ∑_73_PAHs at coastal Deception and Livingston Islands averaged 9.4 (2.9–27.0) ng L^–1^ and 1.9 (0.2–4.5) ng L^–1^, respectively. These concentrations are within the range of those reported in the open oceans and the Arctic Ocean.^3,4,44,45^ However, these coastal concentrations are significantly lower than those reported in other coastal regions such as the coastal eastern Indian Ocean (West Australia coast),^4^ coastal Mediterranean,^46,47^ or Eastern Asia (Eastern China coast).^48−50^ This is consistent with the small population in the research stations at South Bay and Port Foster (Livingston and Deception Islands, respectively) and the remoteness to populated regions. The Antarctic circumpolar current acts as a barrier for the North–South transport of organic pollutants by oceanic currents;^51^ therefore, PAHs reaching coastal Antarctica are driven by long-range atmospheric transport followed by air–water diffusive exchange or wet deposition.^1,4,6,52^

The water-dissolved-phase PAH profile was dominated by 3–4 ring PAHs such as phenanthrene, methylphenanthrenes, pyrene, and fluoranthene, among others, consistent with previous works of PAHs in remote oceanic regions, including Antarctica.^3,4,11,44,45^ For the most abundant PAHs, concentrations at Deception were significantly higher than in Livingston (Figure S3). Concentrations between the latter were not significantly different. Even though the dissolved-phase concentrations were sampled at South Bay during two austral summers, there are some differences between the two years. The Livingston 2015 campaign summer was characterized by an important snowpack accumulated during the previous winter and spring, which melted during the summer. On the contrary, snow deposition was lower in the winter before the Livingston 2018 campaign, with less snow cover and thus lower snowmelt inputs during the summer (see pictures in Figure S1). It has been shown that snow amplification of concentrations and melting is an important input of PAHs during the austral summer, increasing the coastal seawater concentrations of PAHs and other pollutants.^6,53,54^ The concentrations at the end of the Livingston 2015 campaign were slightly higher than those in the 2018 campaign for some alkyl-PAHs, such as dimethylphenanthrenes. During the austral summer of 2015, dissolved- and gas-phase concentrations were close to equilibrium, with a net deposition and net volatilization in the early and late summer, respectively.^6^ During the Livingston 2018 campaign, there were probably, as well, close to equilibrium air and water phase concentrations, as dissolved-phase concentrations were similar in both campaigns. Therefore, the differences in atmospheric deposition between Livingston 2015 and 2018 were due to the wet deposition of snow. Nevertheless, despite these differences in inputs due to snow melting, the overall PAH concentrations in 2015 and 2018 were not different, suggesting that the removal processes of dissolved-phase PAHs could have mitigated differences in the source strength. This mitigation can be caused by the biological pump (sorption to phytoplankton, zooplankton, bacteria, and organic detritus followed by the sinking of particles) or abiotic or biotic degradation. Both processes will affect high and low MW PAHs, respectively (Table S8).

Plankton-phase concentrations of ∑_73_PAHs at coastal Deception and Livingston Islands averaged 495 (72–2005) ng g^–1^ and 102 (15–479) ng g^–1^, respectively. These concentrations are comparable to those reported in the south Atlantic or south Pacific oceans but slightly lower than those reported for the Indian Ocean or the Mediterranean Sea.^11,44^ The PAH profile is similar to that found in the water-dissolved phase, consistent with dynamic plankton–water partitioning, and is dominated by 3–4 ring PAHs, with high abundances of alkyl-PAHs (Figure S2). There is high variability in plankton-phase concentrations, consistent with previous studies,^11,44^ which is probably the result not only of the intrinsic variability of plankton biomass but also of different strengths of sources and degradation processes affecting the PAH concentrations (see below). The comparison of PAH concentrations in plankton for the three campaigns shows that for most PAHs with 3–5 aromatic rings, the levels at Deception Island were significantly higher than those at Livingston Island (Figure S4). For most compounds, there are no significant differences between the two campaigns performed at Livingston Island, despite the higher PAH fluxes from snow melting during the Livingston 2015 campaign.

### Geochemical Evidences for PAH Biodegradation

There are a number of trends and patterns that provide geochemical insights into the biodegradation of PAHs. These include PAH patterns in seawater and plankton, partitioning among the different phases, and the ratios of individual PAHs that can be used to identify either the source of PAHs or the extent of weathering (photo- and biodegradation). First, biodegradation is faster for parent PAHs than alkyl-PAHs.^55^ We found that Phe/(∑MPhe + ∑DMPhe + ∑TMPhe), DBT/(∑MDBT + ∑DMDBT), and Pyr/(∑MPyr + ∑DMPyr) ratios in the water-dissolved phase were significantly lower for the Livingston 2015 campaign than in the other two campaigns. While these ratios were kept consistently low during the Livingston 2015 campaign, the ratios decreased over the austral summer for the other two campaigns, but these never reached the low levels of the Livingston 2015 campaign (Figures S5). Second, generally, LMW PAHs (having 3–4 aromatic rings) are mainly found in the dissolved phase, are more bio-available for degradation, and show higher degradation rates.^56−58^ Thus, the ratios between LMW and HMW PAH are good indicators of the degree of biodegradation of PAH.^11^ These were lower for the Livingston 2015 campaign than for the other two campaigns, even though a decrease in this ratio during the Livingston 2018 campaign was observed (Figure S6).

Third, photodegradation could occur in surface waters. Photolysis of B[*a*]Ant has been shown to be faster than that of Cry, and thus the ratio B[*a*]Ant/Cry can be tentatively used for photodegradation.^59^ The B[*a*]Ant/Cry ratio in the dissolved-phase samples from the surface was lower as well during the 2015 campaign than the other two campaigns, also consistent with a higher degree of photodegradation. However, the extent of photodegradation was discernible only at the surface since such a trend of the B[*a*]ant/Cry ratio was not observed for the plankton-phase PAHs integrating the water column. A larger sink due to biodegradation than photodegradation has also been reported before for other oceanic regions.^11^

Finally, an assessment of partitioning also provides additional clues on the relevance of biodegradation. The PAH partition between the dissolved phase and the organic matter pools due to their hydrophobicity. Compound-specific bioconcentration factors (BCF, L kg^–1^) in all of the samples were calculated by
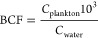
1where *C*_plankton_ is the concentration in the plankton phase (ng g^–1^) and *C*_water_ is the concentration in the water-dissolved phase (ng L^–1^).

There were significant linear correlations between the BCFs and the octanol–water partition coefficient (*K*_OW_) for both the Deception 2017 and Livingston 2018 campaigns (*p* < 0.05), explaining 28 and 39% of the variability in BCFs. However, for the Livingston 2015 campaign, even significantly, it only explained 4% of the variability. Such differences can be due to various factors, such as faster growth rates of the microbial communities favored by snow inputs, thus inducing lower *C*_plankton_ by dilution. Alternatively, a larger degradation of dissolved-phase compounds (lower *C*_water_) can increase the BCF of the less hydrophobic chemicals, resulting in shallower slopes during the 2015 campaign and thus driving a lower degree of equilibrium between the dissolved and plankton phases. (Figure S7).

Further evidence of PAH degradation from partitioning comes from a multicompartmental work comprising atmospheric, snow, soil, and seawater samples for the Livingston 2015 campaign.^54^ In this companion work, it was observed that the fugacity ratios between seawater and air for alkylated PAHs were higher, closer to the predicted values due to inputs from snow amplification than for parent PAHs. Such differences between parent and alkyl-PAHs are also indicative of microbial degradation of PAH. In addition, a comprehensive assessment of the variability of plankton-phase PAHs from various oceans (including the Livingston 2015 concentrations)^11^ showed that biodegradation was the main sink for 3–4 ring PAHs.

All of these geochemical pieces of evidence show a key role of biodegradation explaining the occurrence of PAHs, but with different strengths for different austral summers and of larger magnitude during the Livingston 2015 campaign. Whereas the increasing trend in PAH concentrations for the 2015 campaign (Figure 2) was driven by the large PAH inputs from snow melting, geochemical evidence suggests that biodegradation was more active during this austral summer (2015) than for the other campaigns (2017, 2018). The importance of biodegradation on the PAH occurrence is hidden under the increase of PAH due to snowmelt inputs. Conversely, for the 2017 and 2018 campaigns, when the snow had already melted at the beginning of the campaign (smaller snowpack), the decrease of the PAH concentrations was apparent (Figure 2), consistent with biodegradation, even if it was of lower magnitude than during the 2015 campaign. This suggests that the biogeochemistry of PAHs in coastal Antarctica is not only due to different magnitudes of snow-related inputs but also to different extents of biodegradation driven by potential differences in the HCB community, which in turn would also be dependent on the snow- and glacier-derived inputs of nutrients or organic matter.^60^ In fact, the concentrations of inorganic nutrients during the Livingston 2015 campaign were significantly higher than the other two campaigns (Figure S8).

### Microbial Structure and Hydrocarbonoclastic Bacteria Abundance

Characterization of the free-living microbial communities was performed for concurrent samples to those of PAHs and for the three sampling campaigns and is reported here for the first time. The microbial community composition was significantly different in each campaign (Permanova test, *p* < 0.05), and samples clustered together according to the campaigns (Figure S9). In general, microbial communities in all three campaigns were dominated by the classes *Bacteroidia* (ranging from 10 to 53% of relative contribution to the total 16S rDNA pool, mean 38 ± 9%), *Gammaproteobacteria* (9–71, 30 ± 11%), and *Alphaproteobacteria* (15–60%, mean 31 ± 11%) (Figure S10). *Bacteroidia* was mostly accounted for by the order *Flavobacteriales*, *Gammaproteobacteria* by orders *Enterobacterales* and *Pseudomonadales*, and *Alphaproteobacteria* by *SAR11* and *Rhodobacterales*, consistent with previous reports in this region.^24,61,62^ Within *Alphaproteobacteria*, *SAR11* had a significantly higher contribution in Deception 2017, where the lowest nutrient concentrations were measured, consistent with the *SAR11* frugal lifestyle and their success in nutrient-poor environments.^63^ Within *Gammaproteobacteria*, *Enterobacterales* and *Pseudomonadales* had a significantly higher contribution in Livingston 2015 site with the highest concentrations of nutrients, whereas *Gammaproteobacteria* other than *Pseudomonadales* and *Enterobacterales* showed the opposite pattern. This is consistent with their copiotroph lifestyle already observed in other marine environments.^64^ Within *Bacteroidia*, *Flavobacteriales* was the main taxa for the three campaigns, with a decrease in Deception 2017 in favor of *SAR11* compared to the other campaigns.

**Figure 3:**
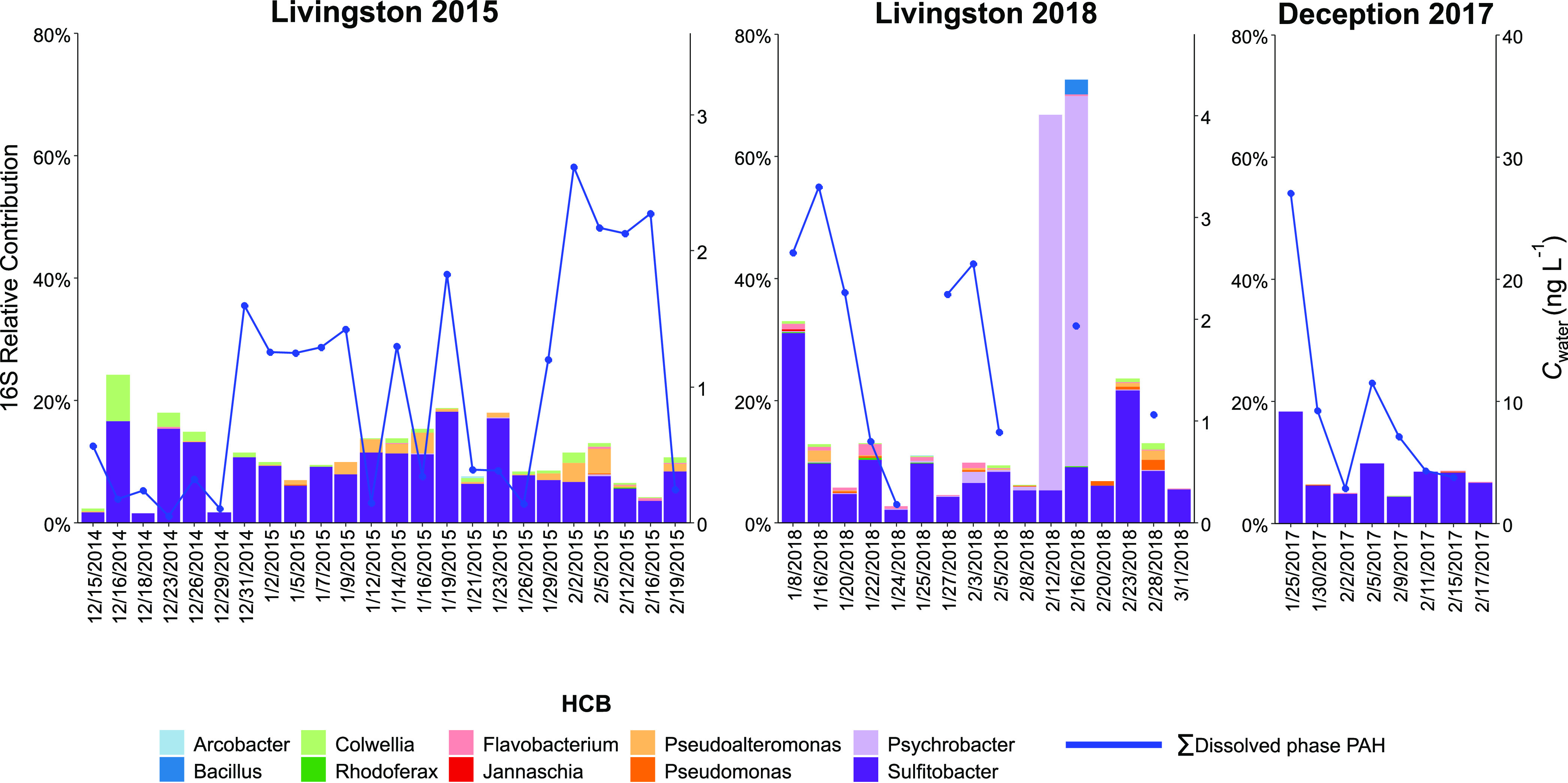
Relative contributions of the top 10 more abundant hydrocarbonoclastic bacteria (HCB) found in coastal waters of Livingston and Deception Islands for the three sampling campaigns.

Bacterial taxa identified as HCB contributed to the total pool of 16S ASV reads on average 13 ± 13%, ranging from 2 to 30% (except for 2 days at Livingston 2018 when a bloom of *Psychrobacter* reached a contribution of 58%) (Figure 3). The lowest diversity of HCB taxa was detected at Deception 2017, whereas the HCB community in Livingston Island was richer and more diverse (Table S9). HCB was mostly accounted for by *Sulfitobacter* at the genus level at all of the campaigns, with relative abundances ranging from 0.015 to 30%, with mean values of 9 ± 6%. The presence of *Sulfitobacter* in the Antarctic Peninsula has also been reported previously at similar relative abundances.^65,66^ Other major contributors were facultative HCB such as *Psychrobacter*, *Pseudoalteromonas*, *Alkanindiges*, and *Acinetobacter*. Obligate HCB (*Oleispira*, *Colwellia*)^25^ and other facultative HCB such as *Marinomonas*, *Glaciecola*, and *Pseudomonas* were present at lower relative abundances (Table S9). These HCB taxa have been previously found in Antarctic waters and sediments, and in some manipulative experiments at low temperatures, their role in PAH degradation has been reported.^16,40,67^

The relative abundances of 16S rDNA ASV reads calculated using 16S amplicon sequencing data were validated for genus *Colwellia* by qPCR using taxonomical specific primers. A significant correlation between the relative abundances of *Colwellia* in the 16S rDNA ASV pool vs. absolute concentrations of *Colwellia* assessed by qPCR was observed (*p* < 0.05) in samples obtained at Johnsons Dock during the Livingston 2015 campaign (Figure S11). These results provide a quantitative validation of the relative abundances assessed by 16S sequencing.

### Role of Bacteria on the Fate and Occurrence of PAHs

**Figure 4:**
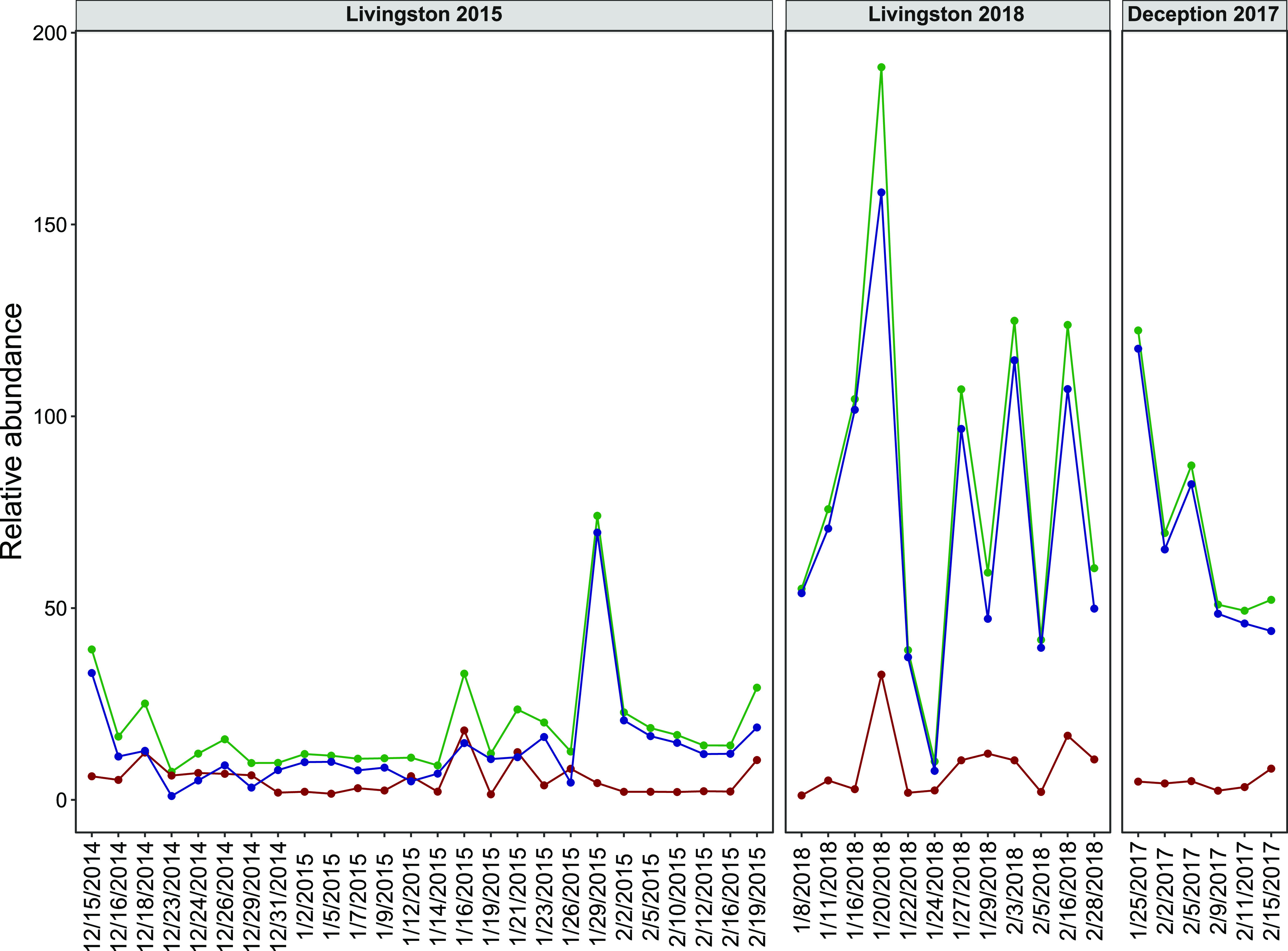
Temporal trends of relative abundances of PAHs in the water-dissolved phase after normalizing to chrysene for the three campaigns. The benchmarking using chrysene blocks the influence of different inputs such as snow, allowing us to compare the different degradation for the three campaigns, with lower relative contributions at Livingston 2015 consistent with larger biodegradation. The green line shows the sum of the relative abundances of all of the PAHs, the blue line is the sum of LMW PAHs, and the red line is the sum of HMW PAHs.

As discussed above, the temporal trends of PAHs in water and plankton, as well as the diagnostic ratios, showed evidence of the influence of varying snow-associated inputs of PAHs and degradation. Benchmarking allows blocking such variability in PAH abundances due to processes (inputs or sinks) different than biodegradation. Such an approach has been used before for assessing persistence in field and laboratory works.^68,69^ Here, we used chrysene as a reference chemical for the benchmarking; since it is not within the 3–4 aromatic ring PAHs, which are efficiently biodegraded, it is degraded by photolysis at a slower pace than other PAHs, and it is still not very hydrophobic, so it does not settle fast to sediments. Conversely, chrysene is also abundant in snow inputs,^6,11^ and benchmarking by chrysene allows blocking the variable yearly influence of snow inputs. The temporal trends of relative abundances of PAHs in the water-dissolved phase (as those derived by normalizing to chrysene) showed that the increase of concentrations during the Livingston 2015 campaign due to snow inputs was not apparent anymore (Figure 4). The lower relative abundances of chrysene-normalized LMW PAH concentrations during Livingston 2015 support a higher PAH biodegradation in that period. The potential for biodegradation was assessed by the presence of HCB that was, however, not significantly higher in Livingston 2015 than in the other 2 campaigns (Figure 3), suggesting that activity is not directly uniquely linked to HCB abundances but to other environmental parameters such as temperature and nutrient availabilities,^70^ factors that are related to snow/glacier inputs, which can induce a different composition of HCB in different years.

**Figure 5:**
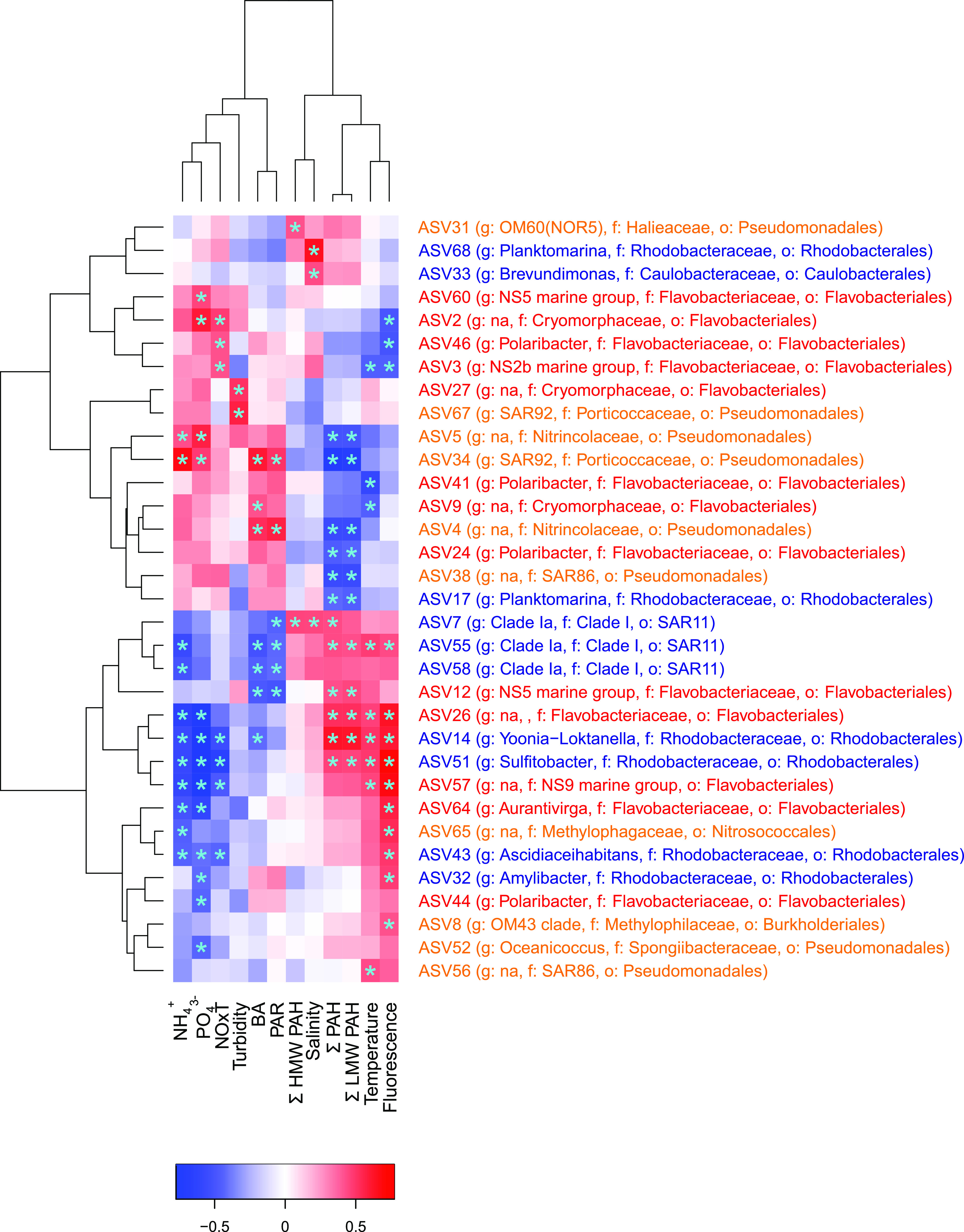
Correlation heatmap between ASV relative abundances and environmental variables. PAH concentrations in the water-dissolved phase were benchmarked for chrysene. Positive and negative correlations are indicated with red and blue colors, respectively. Significant correlations (*p* < 0.05) are represented with a blue asterisk. NH_4_^+^, PO_4_^3–^, NOxT: Inorganic nutrients. BA: Bacterial abundance. PAR: Photosynthetically active radiation. Colors on the ASV taxonomy indicate the class (*Gammaproteobacteria* in yellow, *Alphaproteobacteria* in blue, and *Bacteroidia* in red).

To get insights into taxa in the HCB group with a potential relevance on PAH fate, Pearson correlations between the relative abundances of specific taxa and the chrysene-normalized PAH concentrations in the water-dissolved phase were calculated. These correlations were significantly positive and negative for several taxa (Figure 5). The significant positive correlations may be related to common factors driving the occurrence of both abundances (for example, glacier/snow melting inputs) and do not offer evidence for causation between bacterial abundances and PAHs in either direction. On the other hand, there were significant negative correlations between the abundance of LMW PAHs (and thus total PAHs) and that of 6 specific ASV, four of which belonged to *Pseudomonadales*. This group is described as facultative HCB in many studies and they bloom after pulses of PAH in the early stages of oil spill accidents in seawater at low temperatures.^71−73^ At low “background” concentrations of PAH, *Pseudomonadales* have been found to play a role in degrading PAH at the surface microlayer of coastal Antarctica.^16^ That the 3–4 aromatic ring PAHs decrease when the abundances of *Pseudomonadales* increase is consistent with their microbial degradation. Interestingly, water-dissolved-phase HMW PAHs did not show any significant negative correlations with the 16S amplicon sequencing data, probably because these included free-living bacteria but not the particle-associated bacteria which may interact with particle-bound HMW PAHs.^16^ PLS analysis showed that members of *Pseudomonadales* and *Flavobacteriales* orders were key contributors to principal component 1 (Figures S12 and S13), which separated the Livingston 2015 campaign from the others. It is noteworthy that these same ASV of *Pseudomonadales* and *Flavobacteriales* were those correlating negatively with PAH concentrations. The degradation of PAHs during this campaign was maximal, as discerned above from geochemical evidence including the PAH diagnostic ratios. High nutrient conditions, maybe due to a stronger glacier/snow melting, may have favored the higher abundances of these taxa. If true, this would mean that snow melting triggers a higher PAH input but also a larger biodegradation potential of the microbial community.

The concurrent assessment of PAHs during three austral summers shows a large yearly variability in the occurrence of certain PAHs. The importance of snow inputs was highly variable, with a clear influence on the temporal trends of PAHs. However, biodegradation was also maximal for the same year with large inputs of melting snow. There was a covariability of certain bacteria and PAHs that would need further research to confirm their role as keystone taxa of microbiomes degrading PAHs in polar environments. The concurrent characterization of pollutants and microbial communities in field-derived time series of measurements is a powerful approach and provides important tools to discern the complexity of biogeochemistry and thus the fate and sinks for pollutants in the environment.

## Supporting Information Available

Analytical procedures for PAH determination; quality assurance/quality control for PAH analysis; description and coordinates of the sampling stations; ancillary data for the seawater samples; recoveries of PAH surrogates for each sampling campaign; limits of quantification for PAHs for seawater and plankton samples; nutrient concentrations and bacterial abundances for the water samples; individual PAH concentrations in the dissolved- and plankton-phase concentrations; classification of the analyzed PAHs as low molecular weight and high molecular weight; relative abundancies of hydrocarbonoclastic taxa; picture of South Bay for the 2015, 2017, and 2018 campaigns (January) showing the difference in snow cover; occurrence of individual PAHs in plankton for the three sampling campaigns; differences in individual PAH concentrations in the water-dissolved phase and in plankton for the three sampling campaigns assessed by ANOVA followed by a Tukey test; temporal series of the parent/alkyl-PAHs concentration ratios and LMW/HMW ratios in the water-dissolved phase for the three campaigns; data on bioconcentrations factors versus octanol–water partition coefficients; concentrations of *in situ* inorganic nutrients; nonmetric multidimensional scaling (NMDS) of the microbial communities composition; correlation plots between amplicon sequencing relative abundance and qPCR relative abundance for the genus Colwellia at Jhonsons Dock; and partial least squares regression (PLS) and weight of the VIP x variables (individual ASV) over components 1 and 2 of the PLS (PDF)
