# Supplementary material for: Snow-Dependent Biogeochemical Cycling of Polycyclic Aromatic Hydrocarbons at Coastal Antarctica
Source: Environ Sci Technol. 2023 Jan 19;57(4):1625–36. doi: 10.1021/acs.est.2c05583 (PMC9893724; doi:10.1021/acs.est.2c05583)
Supplement: Supplementary file 1 — es2c05583_si_001.pdf [file es2c05583_si_001.pdf]

SUPPORTING INFORMATION

Snow-Dependent Biogeochemical Cycling of Polycyclic Aromatic Hydrocarbons at Coastal Antarctica

Jon Iriarte, Jordi Dachs*, Gemma Casas, Alicia Martínez-Varela, Naiara Berrojalbiz and Maria Vila-Costa*

Department of Environmental Chemistry, Institute of Environmental Assessment and Water Research, IDAEA-CSIC; 08034 Barcelona, Catalunya, Spain

Summary: 39 pages. 2 annexes, 9 tables, 13 figures

Table of Contents

Annex S1. **Analytical Procedures for PAH determination**

Annex S2. Quality Assurance/Quality Control for PAH analysis

Table S1. Description and coordinates of the sampling stations.

Table S2. Ancillary data for the seawater samples (average between 0.2 to 5 m depth) obtained from the CTD cast.

Table S3. Recoveries of PAH surrogates for each sampling campaign.

Table S4. Limits of quantification for PAHs for seawater and plankton samples for Deception 2017 and Livingston 2018.

Table S5. Nutrient concentrations and bacterial abundances for the water samples.

Table S6. Individual PAH concentrations in the dissolved phase (ng L^{-1}) for the Deception 2017 and Livingston 2018 campaigns.

Table S7. Individual PAH Plankton phase concentrations (ng g_{dw}^{-1}) for the Deception 2017 and Livingston 2018 campaigns.

Table S8. Classification of the PAHs analyzed in this study as low molecular weight (LMW) PAH or high molecular weight (HMW) PAH.

Table S9. Relative abundances (%) of HCB taxa, expressed as the mean, standard deviation, maximum and minimum of each taxa for each campaign.

Figure S1. Picture of South bay for the 2015, 2017 and 2018 campaigns (January) showing the difference in snow cover.

Figure S2: Occurrence of individual PAHs in plankton for the three sampling campaigns.

Figure S3: Differences in individual PAH concentrations in the water dissolved phase for the three sampling campaigns assessed by an ANOVA followed by a Tukey test.

Figure S4: Differences in individual PAH concentrations in the plankton phase for the three sampling campaigns assessed by an ANOVA followed by a Tukey test.

Figure S5: Temporal series of the parent/alkyl-PAHs concentration ratios in the water dissolved phase for the three campaigns.

Figure S6: Temporal series of the LMW/HMW ratios in the water dissolved phase for the three campaigns.

Figure S7: Bioconcentration factors (Log BCF) versus octanol-water partition coefficients (Log K_{ow}) for the three sampling campaigns.

Figure S8: Boxplot of inorganic nutrient concentrations for the three campaigns.

Figure S9: Non-metric Multidimensional Scaling (NMDS) plot for clustering samples according to their ASV composition.

Figure S10: Structure of the bacterial community for the three sampling campaigns.

Figure S11: Correlation between amplicon sequencing relative abundance and qPCR relative abundance for the genus *Colwellia* at Jhonsons dock.

Figure S12: Partial least squares regression (PLS).

Figure S13: Plot showing the weight of the VIP x variables (individual ASV) over the components 1 and 2 of the PLS.

References

Annex S1. Analytical Procedures for PAH determination

Sample handling (filtrations and elution through the XAD-2 column) was performed outside the Antarctic stations to avoid potential contamination from the in-side laboratory. Further processing of the samples was performed in a clean lab (ISO 5). The analytical methodology has been reported elsewhere. [1] Briefly, XAD-2 columns were eluted with 200 mL of methanol, followed by 200 mL of dichloromethane and 100 ml of hexane at a flow rate of 2 mL min-1 using an axial piston pump. The methanol fraction was concentrated and liquid-liquid extracted with 50 mL of hexane for three times. The hexane extracts were dried over anhydrous sodium sulphate and combined with the dichloromethane and hexane fractions. After reducing the volume with a rotatory evaporator, the extracts were fractioned with 25 ml of hexane, 40 ml of dichloromethane:hexane (1:3, v:v) and 20 ml dichloromethane:acetone (70:30, v:v) on a 5 g of a silica gel column (silica 60-200 mesh, activated at 250 °C for 24 h) and 3 g of 3 % deactivated neutral alumina column (aluminum oxide 90, activated at 250 °C for 12 h). Plankton samples were lyophilized during 5 h and Soxhlet-extracted with dicloromethane:hexane (1:1, v:v) during 24 h, and fractionated using the same procedure than for the dissolved phase. The perdeuterated standards acenaphthene-d10, phenanthrene-d10, crysene-d12 and perylene-d12 were added before extraction for recovery control.

Before the injection, the vials were spiked with 50 ng of the perdeuterated standards anthracene-d10, pyrene-d10 and benzo[b]fluoranthrene-d12 that were used as injection (internal) standards. PAH quantification was performed with an Agilent 6890 Series gas chromatograph coupled to a mass spectrometer Agilent 5973 (GCMS) operating in selected ion monitoring (SIM) and electron impact mode (EI). A 30 m capillary column (HP-5MS, 0.25mm x 0.25um film thickness) was used. The oven temperature was increased to 90 °C (held for 1 min), then increased to 175 °C at 6 °C/min (held for 4 min), increased to 235 °C at 3 °C/min, increased to 300 °C at 8 °C/min (held for 8 min), and finally to 315 °C for 5 min (held for 8 min). Injector and transfer line temperatures were 280 and 300 °C, respectively. 2 µl of sample were injected in split less mode.

Annex S2. Quality Assurance/Quality Control for PAH analysis

All containers, tubes and connections used during sampling and chemical analysis were of stainless steel, glass or PTFE, and they had been pre-cleaned with acetone prior use in order to avoid contamination.

Field blanks consisted of GF/D filters and XAD-2 columns that followed the same process as the samples albeit without the pass of plankton or water. Procedural and/or field blanks were analyzed with each batch of 4-6 samples to monitor potential contamination during sampling and extraction. The limits of quantification (LOQs) were defined as the mean concentration of field or procedural blanks, whichever higher, plus three times the standard deviation of the blank response. For the analytes which were not found in procedural blanks, LOQ were derived from the lowest standard in the calibration curve (three times the signal-to-noise ratio of the lowest standard in the calibration curve). Surrogate recoveries are resumed in Table S3 and limits of quantification are listed in Table S4.

Table S1. Description and coordinates of the sampling stations.

Sampling station	Campaign	Sample type	S	W	Sampling name	Plankton net depth
St-1	Livingston 2018	Seawater and plankton	62º 38.792'	60º 38.411'	Hannah Point	30 m
St-2	Livingston 2018	Seawater and plankton	62º 38.346'	60º 23.912'	South Bay	30 m
St-3	Livingston 2015	Seawater and plankton	62º 39.425'	60º 23.277'	Raquelia Rocks	30 m
St-4	Livingston 2015 & 2018	Seawater and plankton	62º39,556’	60º 22,132’	Johnsons Dock	13-14 m
St-5	Livingston 2018	Seawater and plankton	62º 37.718'	60º 21.400'	Emona Anchorage	30 m
St-6	Livingston 2018	Plankton	62º 42.694'	60º 26.547'	Miers Bluff	60 m
St-7	Livingston 2018	Plankton	62º 41.757'	60º 20.008'	False Bay	40 m
St-8	Deception 2017	Seawater and plankton	62º 59.386'	60º 37.115'	Colatinas Point	15 m

Table S2. Ancillary data for the seawater samples (average between 0.2 to 5 m depth) obtained from the CTD cast. PAR means photosynthetic active radiation and n.d. means no-data available.

Date	Sampling station	Campaign	Temperature (°C)	Salinity (PSU)	Fluorescence (RFU)	PAR	Turbidity (NTU)
15/12/2014	St-3	Livingston 2015	0.46	34.01	0.52	129.51	4.86
16/12/2014	St-4	Livingston 2015	0.82	33.68	0.33	161.21	5.97
18/12/2014	St-3	Livingston 2015	0.70	33.81	0.68	207.84	5.50
23/12/2014	St-4	Livingston 2015	1.24	33.70	0.50	203.10	3.90
24/12/2014	St-3	Livingston 2015	0.20	34.00	0.22	367.00	3.17
26/12/2014	St-4	Livingston 2015	1.24	33.70	0.50	203.10	3.90
29/12/2014	St-3	Livingston 2015	0.48	33.99	0.41	295.60	3.59
31/12/2014	St-4	Livingston 2015	1.33	33.84	0.28	431.20	3.92
02/01/2015	St-4	Livingston 2015	1.52	33.77	0.25	294.90	2.65
05/01/2015	St-3	Livingston 2015	1.34	33.55	0.59	259.30	4.87
07/01/2015	St-4	Livingston 2015	1.32	33.59	0.43	550.20	4.92
09/01/2015	St-3	Livingston 2015	1.12	33.95	0.57	126.04	2.27
12/01/2015	St-3	Livingston 2015	1.64	33.81	0.43	307.60	3.13
14/01/2015	St-4	Livingston 2015	1.97	33.36	1.21	154.07	3.68
16/01/2015	St-3	Livingston 2015	1.26	33.91	1.91	68.30	3.02
19/01/2015	St-4	Livingston 2015	1.23	33.69	1.01	176.72	5.95
21/01/2015	St-3	Livingston 2015	0.40	33.55	0.91	179.76	5.70
23/01/2015	St-4	Livingston 2015	1.15	33.34	1.57	49.63	6.65
26/01/2015	St-3	Livingston 2015	1.55	33.55	1.12	81.98	5.79
29/01/2015	St-4	Livingston 2015	1.51	33.29	1.63	29.66	10.55
02/02/2015	St-3	Livingston 2015	1.82	33.73	1.21	67.37	5.07
05/02/2015	St-4	Livingston 2015	1.71	33.19	1.04	67.40	12.38
10/02/2015	St-3	Livingston 2015	1.66	33.58	1.62	46.30	7.24
12/02/2015	St-4	Livingston 2015	1.83	33.33	1.52	42.01	10.46
16/02/2015	St-3	Livingston 2015	1.82	33.52	0.68	305.40	6.15
19/02/2015	St-4	Livingston 2015	1.91	33.39	0.92	137.83	8.23
25/01/2017	St-8	Deception 2017	2.55	34.00	5.32	10.22	3.96
30/01/2017	St-8	Deception 2017	2.01	34.02	1.27	267.22	3.66
02/02/2017	St-8	Deception 2017	2.23	33.48	1.03	236.03	3.64
05/02/2017	St-8	Deception 2017	2.40	33.73	4.21	102.64	4.35
09/02/2017	St-8	Deception 2017	2.43	33.74	4.31	17.63	3.68
11/02/2017	St-8	Deception 2017	2.71	33.68	5.46	52.19	4.13
15/02/2017	St-8	Deception 2017	2.43	33.81	2.36	57.16	3.67
17/02/2017	St-8	Deception 2017	2.50	33.90	1.29	202.91	3.67
08/01/2018	St-4	Livingston 2018	n.d	n.d	n.d	n.d	n.d
11/01/2018	St-2	Livingston 2018	n.d	n.d	n.d	n.d	n.d
16/01/2018	St-4	Livingston 2018	n.d	n.d	n.d	n.d	n.d
20/01/2018	St-2	Livingston 2018	1.60	34.14	0.66	0.27	3.56
22/01/2018	St-4	Livingston 2018	1.42	33.30	0.40	0.27	7.84
23/01/2018	St-6	Livingston 2018	0.68	33.63	0.29	0.27	7.17
23/01/2018	St-7	Livingston 2018	1.99	33.96	0.59	0.27	3.74
24/01/2018	St-2	Livingston 2018	2.23	33.94	0.88	0.27	3.66
25/01/2018	St-4	Livingston 2018	1.71	33.46	0.27	0.27	5.85
27/01/2018	St-2	Livingston 2018	2.43	33.68	0.31	0.27	4.86
29/01/2018	St-4	Livingston 2018	1.87	33.49	0.58	0.27	6.04
03/02/2018	St-2	Livingston 2018	1.59	34.75	0.15	0.27	3.55
05/02/2018	St-4	Livingston 2018	1.47	33.61	0.44	0.27	6.41
08/02/2018	St-1	Livingston 2018	2.25	33.82	0.19	0.27	8.56
12/02/2018	St-2	Livingston 2018	1.76	33.67	0.18	0.27	7.11
16/02/2018	St-4	Livingston 2018	1.78	33.26	0.23	0.27	7.44
20/02/2018	St-2	Livingston 2018	2.93	33.86	0.23	0.27	3.87
23/02/2018	St-4	Livingston 2018	1.82	33.16	0.19	0.27	9.83
28/02/2018	St-5	Livingston 2018	1.14	33.63	0.28	0.27	5.44

28/02/2018	St-2	Livingston 2018	n.d	n.d	n.d	n.d	n.d
01/03/2018	St-1	Livingston 2018	1.73	33.94	0.30	0.27	4.08

Table S3. Recoveries of PAH surrogates for each sampling campaign.

Livingston 2015

Surrogate	Seawater	Plankton
acenaphthene-d10	63±9.5%	65±18%
phenanthrene-d10	96±17%	72±12%
crysene-d12	85±25%	85±14%
perylene-d12	100±17%	100±25%

Livingston 2018

Surrogate	Seawater	Plankton
phenanthrene-d10	19±12%	51±5.7%
perylene-d12	67±33%	77±23%

Deception 2017

Surrogate	Seawater	Plankton
phenanthrene-d10	20±11%	31±4.8%
perylene-d12	64±32%	66±7.5%

Table S4. Limits of quantification for PAHs for seawater and plankton samples for Deception 2017 and Livingston 2018. The limits of quantification (LOQs, ng) were defined as the mean concentration of field blanks plus three times the standard deviation of the blank response. For the analytes not detected in blanks, LOQs were derived from the lowest standard in calibration curve (three times the signal-to-noise ratio of the lowest standard in the calibration curve).

PAH	Liv. 2018 seawater (ng)	Dec. 2017 seawater (ng)	Liv. 2018 plankton (ng)	Dec. 2017 plankton (ng)
MFlu(1)	0.005	0.005	0.005	0.018
MFlu(2)	0.005	0.005	0.005	0.021
MFlu(3)	0.005	0.005	0.018	0.038
MFlu(4)	0.058	0.005	0.040	0.066
DBT	0.069	0.086	0.077	0.011
MDBT(1)	0.068	0.137	0.020	0.011
MDBT(2)	0.034	0.101	0.009	0.005
MDBT(3)	0.005	0.043	0.010	0.005
DMDBT(1)	0.005	0.085	0.005	0.005
DMDBT(2)	0.005	0.044	0.005	0.005
DMDBT(3)	0.042	0.189	0.005	0.005
DMDBT(4)	0.005	0.095	0.005	0.005
DMDBT(5)	0.005	0.037	0.005	0.005
Phe	0.632	0.941	0.244	0.364
MPhe(1)	0.065	0.108	0.024	0.047
MPhe(2)	0.084	0.157	0.035	0.064
MPhe(5)	1.017	1.085	0.009	0.005
MPhe(3)	0.085	0.136	0.034	0.087
MPhe(4)	0.045	0.131	0.028	0.057
DMPhe(1)	0.016	0.051	0.005	0.005
DMPhe(2)	0.017	0.039	0.005	0.005
DMPhe(3)	0.005	0.005	0.005	0.005
DMPhe(4)	0.047	0.129	0.005	0.005
DMPhe(5)	0.029	0.070	0.005	0.005
DMDBT(6)	0.005	0.005	0.005	0.005
DMPhe(6)	0.023	0.065	0.005	0.022
DMPhe(7)	0.005	0.005	0.005	0.005
DMPhe(8)	0.005	0.005	0.005	0.005
DMPhe(9)	0.009	0.005	0.005	0.005
DMPhe(10)	0.005	0.005	0.005	0.005
TMPhe(1)	0.005	0.005	0.005	0.005
TMPhe(2)	0.005	0.005	0.005	0.005
TMPhe(3)	0.005	0.005	0.005	0.005
TMPhe(4)	0.005	0.005	0.005	0.005
TMPhe(5)	0.005	0.005	0.005	0.005

TMPhe(6)	0.005	0.005	0.005	0.005
TMPhe(7)	0.005	0.005	0.005	0.005
TMPhe(8)	0.005	0.005	0.005	0.005
TMPhe(9)	0.005	0.005	0.005	0.005
TMPhe(10)	0.005	0.005	0.005	0.005
TMPhe(11)	0.005	0.005	0.005	0.005
TMPhe(12)	0.005	0.005	0.005	0.005
Ant	0.580	0.049	0.019	0.026
Flt	0.581	1.761	0.036	0.253
Pyr	0.981	1.085	0.031	0.079
MPyr(1)	0.005	0.005	0.005	0.005
MPyr(2)	0.092	0.124	0.005	0.005
MPyr(3)	0.063	0.037	0.005	0.005
MPyr(4)	0.087	0.093	0.005	0.005
MPyr(5)	0.064	0.005	0.005	0.005
DMPyr(1)	0.005	0.005	0.005	0.005
DMPyr(2)	0.005	0.005	0.005	0.005
DMPyr(3)	0.005	0.005	0.005	0.005
DMPyr(4)	0.005	0.005	0.005	0.005
DMPyr(5)	0.005	0.033	0.005	0.005
DMPyr(6)	0.005	0.035	0.005	0.005
DMPyr(7)	0.005	0.055	0.005	0.005
DMPyr(8)	0.005	0.147	0.005	0.005
B[g,h,i]f	1.263	1.466	0.160	0.160
B[a]ant	0.160	0.160	0.160	0.160
Cry	0.160	0.160	0.160	0.160
MCry(1)	0.160	0.160	0.160	0.160
MCry(3)	0.160	0.160	0.160	0.160
MCry(4)	0.160	0.160	0.160	0.160
MCry(2)	0.160	0.160	0.160	0.160
B[b]f	0.035	0.090	0.134	0.010
B[k]f	0.072	0.118	0.010	0.010
B[e]pyr	0.160	0.160	0.160	0.160
B[a]pyr	0.160	0.160	0.160	0.160
Pery	0.160	0.160	0.160	0.160
In[1,2,3-cd]pyr	0.160	0.430	0.160	0.160
Dib[a,h]ant	0.160	0.160	0.160	0.160
B[g,h,i]pery	0.160	0.160	0.160	0.160

Table S5: Nutrient concentrations and bacterial abundances for the water samples. Bacterial abundances were measured by flow cytometry for the Deception 2017 and Livingston 2018 campaigns, but by counts of the 16S for the Livingston 2015 campaign. Those measured by flow cytometry are multiplied by 1000 to transform the units from cells/mL to copies of 16S/L, assuming that each cell has 1 copy of the 16S gene.

Date	Station	Campaign	NO_3^- + NO_2^- (µmol L^{-1})	N-NH_4^+ (µmol L^{-1})	P-PO_4^{3-} (µmol L^{-1})	Bacterial abundance (copies16S L^{-1})
15/12/2014	St-3	Livingston 2015	29.13	1.34	2.03	2.08E+09
16/12/2014	St-4	Livingston 2015	24.84	3.77	1.74	3.54E+09
18/12/2014	St-3	Livingston 2015	16.18	2.07	1.44	1.25E+09
23/12/2014	St-4	Livingston 2015	23.46	3.94	1.78	6.86E+09
24/12/2014	St-3	Livingston 2015	24.02	4.68	1.65	6.60E+09
26/12/2014	St-4	Livingston 2015	18.49	12.60	1.46	5.09E+09
29/12/2014	St-3	Livingston 2015	27.67	22.62	1.83	6.34E+09
31/12/2014	St-4	Livingston 2015	24.00	22.40	1.77	1.09E+10
02/01/2015	St-4	Livingston 2015	23.83	10.65	1.86	5.78E+08
05/01/2015	St-3	Livingston 2015	15.39	22.75	1.35	8.08E+09
07/01/2015	St-4	Livingston 2015	17.27	22.48	1.64	5.73E+09
09/01/2015	St-3	Livingston 2015	15.46	22.51	1.35	2.00E+10
12/01/2015	St-3	Livingston 2015	21.49	7.95	1.61	1.23E+10
14/01/2015	St-4	Livingston 2015	14.87	21.41	1.36	1.42E+10
16/01/2015	St-3	Livingston 2015	22.88	8.84	1.64	4.21E+09
19/01/2015	St-4	Livingston 2015	19.51	22.95	1.72	1.64E+10
21/01/2015	St-3	Livingston 2015	25.82	22.64	1.81	5.90E+09
23/01/2015	St-4	Livingston 2015	21.41	1.66	1.75	7.27E+09
26/01/2015	St-3	Livingston 2015	20.18	23.21	1.66	5.70E+09
29/01/2015	St-4	Livingston 2015	14.16	21.76	1.75	1.34E+09
02/02/2015	St-3	Livingston 2015	12.00	22.77	1.72	1.15E+09
05/02/2015	St-4	Livingston 2015	18.36	8.59	1.87	1.33E+09
10/02/2015	St-3	Livingston 2015	21.87	23.15	1.95	4.78E+10
12/02/2015	St-4	Livingston 2015	12.97	22.83	2.19	7.85E+09
16/02/2015	St-3	Livingston 2015	23.92	22.86	1.96	2.98E+09
19/02/2015	St-4	Livingston 2015	20.66	14.48	2.11	1.29E+09
25/01/2017	St-8	Deception 2017	7.65	0.56	0.46	5.12E+08
30/01/2017	St-8	Deception 2017	20.40	0.27	1.08	4.57E+08
02/02/2017	St-8	Deception 2017	18.93	0.11	1.04	4.97E+08
05/02/2017	St-8	Deception 2017	10.13	0.05	0.58	6.85E+08
09/02/2017	St-8	Deception 2017	16.14	0.40	1.20	6.24E+08
11/02/2017	St-8	Deception 2017	14.34	0.06	0.93	4.48E+08
15/02/2017	St-8	Deception 2017	8.50	0.73	1.12	3.80E+08

17/02/2017	St-8	Deception 2017	13.20	0.11	0.91	3.47E+08
08/01/2018	St-4	Livingston 2018	9.58	7.65	1.06	n.d
11/01/2018	St-2	Livingston 2018	n.d	n.d	n.d	n.d
16/01/2018	St-4	Livingston 2018	19.83	10.80	2.12	2.22E+08
20/01/2018	St-2	Livingston 2018	26.15	1.97	1.69	1.89E+08
22/01/2018	St-4	Livingston 2018	19.36	20.91	1.53	2.37E+08
23/01/2018	St-6	Livingston 2018	n.d	n.d	n.d	n.d
23/01/2018	St-7	Livingston 2018	n.d	n.d	n.d	n.d
24/01/2018	St-2	Livingston 2018	26.83	2.65	1.87	2.15E+08
25/01/2018	St-4	Livingston 2018	18.31	2.84	1.42	1.98E+08
27/01/2018	St-2	Livingston 2018	23.27	2.52	1.40	1.47E+08
29/01/2018	St-4	Livingston 2018	n.d	n.d	n.d	1.54E+08
03/02/2018	St-2	Livingston 2018	18.82	10.80	1.65	9.50E+07
05/02/2018	St-4	Livingston 2018	15.32	5.08	1.46	1.50E+08
08/02/2018	St-1	Livingston 2018	23.36	3.07	1.62	1.43E+08
12/02/2018	St-2	Livingston 2018	20.70	13.05	1.33	4.57E+07
16/02/2018	St-4	Livingston 2018	17.75	9.76	1.32	1.68E+08
20/02/2018	St-2	Livingston 2018	20.78	6.97	1.38	n.d
23/02/2018	St-4	Livingston 2018	14.53	8.51	1.26	3.69E+08
28/02/2018	St-5	Livingston 2018	24.25	5.32	1.39	n.d
28/02/2018	St-2	Livingston 2018	18.45	4.72	1.23	n.d
01/03/2018	St-1	Livingston 2018	10.59	1.12	1.16	1.81E+08

Table S6: Individual PAH concentrations in the water dissolved phase (ng L^{-1}) for the Deception 2017 and Livingston 2018 campaigns.

**Date**	25-1-17	30-1-17	2-2-17	5-2-17	9-2-17	11-2-17	15-2-17	8-1-18	11-1-18	16-1-18	20-1-18	22-1-18	24-1-18	27-1-18	29-1-18	3-2-18	5-2-18	16-2-18	28-2-18
**Station**	St-8	St-8	St-8	St-8	St-8	St-8	St-8	St-4	St-2	St-4	St-2	St-4	St-2	St-2	St-4	St-2	St-4	St-4	St-5
**∑MFlu**	0.26	n.q.	0.07	0.82	0.21	0.15	0.08	0.16	0.25	0.15	n.q.	0.03	0.01	0.11	0.05	0.19	0.04	0.09	0.03
**DBT**	0.44	1.49	0.05	0.19	0.11	0.09	0.07	<loq	0.02	0.05	0.04	<loq	<loq	0.02	0.01	0.24	<loq	0.10	0.004
**∑MDBT**	0.41	0.85	0.06	0.30	0.25	0.10	0.09	0.0003	0.04	0.15	0.06	0.0003	0.004	0.04	0.02	0.47	<loq	0.23	0.01
**Phe**	9.37	6.01	0.66	2.40	1.04	0.92	0.62	0.66	0.39	0.50	0.19	0.11	<loq	0.09	0.11	0.45	0.14	0.19	0.11
**∑MPhe**	1.90	<loq	0.21	1.80	0.98	n.q.	0.53	0.31	0.73	0.57	0.79	0.10	0.02	0.66	0.15	0.38	0.14	0.27	0.17
**∑DMPhe**	0.66	0.17	0.16	1.27	0.88	0.37	0.26	0.28	0.50	0.42	0.11	0.10	0.06	0.22	0.08	0.24	0.13	0.24	0.15
**∑TMPhe**	0.14	n.q.	0.03	0.18	0.18	0.09	0.07	0.05	0.08	0.05	n.q.	0.01	0.01	0.02	0.02	n.q.	0.02	0.02	0.02
**Ant**	0.59	0.21	0.03	0.10	0.04	0.03	0.04	<loq	0.11	0.03	<loq	<loq	<loq	<loq	<loq	<loq	<loq	<loq	0.02
**Flt**	7.72	<loq	0.79	1.99	1.30	1.12	0.67	0.35	0.74	0.53	0.20	0.14	<loq	0.35	0.09	0.28	0.13	0.20	0.13
**Pyr**	3.72	<loq	0.50	1.33	1.27	0.84	0.52	0.67	1.06	0.63	0.49	0.22	<loq	0.46	0.15	<loq	0.19	0.23	0.18
**∑MPyr**	0.28	0.03	0.04	0.21	0.21	0.11	0.09	0.06	0.11	0.08	n.q.	0.03	n.q.	0.03	0.03	n.q.	0.02	0.04	0.03
**∑DMPyr**	0.28	<loq	0.04	0.15	0.19	0.09	0.08	0.02	0.09	0.03	n.q.	0.01	0.01	0.02	0.03	0.05	0.01	0.04	0.02
**B[g,h,i]f**	0.53	<loq	0.12	0.38	0.13	0.13	0.39	0.03	0.11	0.04	0.11	0.01	<loq	0.13	0.11	<loq	<loq	0.07	0.12
**B[a]ant**	0.06	<loq	0.01	0.04	0.03	0.02	0.02	0.01	0.11	<loq	0.12	0.00	0.04	0.04	0.04	0.13	0.003	0.04	0.03
**Cry**	0.22	<loq	0.04	0.13	0.14	0.09	0.07	0.05	0.06	0.03	0.01	0.02	0.02	0.02	0.02	0.02	0.02	0.02	0.02
**∑MCry**	0.19	n.q.	n.q.	0.13	0.11	0.07	0.06	0.02	0.05	0.02	n.q.	0.01	n.q.	0.01	0.01	<loq	0.01	n.q.	n.q.
**B[b]f**	0.01	0.07	0.002	0.01	0.002	<loq	0.003	0.001	0.001	0.001	0.001	0.001	<loq	0.001	0.001	<loq	0.001	<loq	0.001
**B[k]f**	0.01	0.08	<loq	0.004	<loq	<loq	<loq	<loq	0.001	0.001	0.002	0.001	<loq	0.002	0.003	<loq	<loq	0.003	0.003
**B[e]pyr**	0.02	n.q.	0.003	0.01	0.01	0.01	0.01	<loq	0.004	0.002	<loq	<loq	n.q.	<loq	<loq	0.02	0.002	<loq	<loq
**B[a]pyr**	0.005	n.q.	<loq	0.002	<loq	<loq	<loq	<loq	<loq	0.005	<loq	<loq	n.q.	<loq	<loq	<loq	<loq	n.q.	<loq
**Pery**	n.q.	0.32	0.003	n.q.	<loq	<loq	<loq	<loq	n.q.	0.002	n.q.	<loq	n.q.	n.q.	n.q.	n.q.	n.q.	n.q.	n.q.
**In[1,2,3-cd]pyr**	0.16	n.q.	0.03	0.03	0.03	0.04	0.07	<loq	0.01	0.02	0.12	0.01	<loq	0.02	0.03	0.06	0.03	0.11	0.03
**Dib[a,h]ant**	n.q.	n.q.	n.q.	n.q.	n.q.	n.q.	n.q.	n.q.	n.q.	n.q.	n.q.	<loq	n.q.	n.q.	n.q.	n.q.	n.q.	n.q.	n.q.
**B[g,h,i]pery**	0.06	n.q.	0.01	0.03	0.01	0.01	0.04	<loq	0.004	<loq	0.03	<loq	<loq	0.005	0.005	<loq	<loq	0.04	0.005

Table S7: Individual PAH Plankton phase concentrations (ng g_{dw}^{-1}) for the Deception 2017 and Livingston 2018 campaigns.

Date	25-1-17	30-1-17	2-2-17	5-2-17	9-2-17	11-2-17	15-2-17	17-2-17	8-1-18	11-1-18	16-1-18	20-1-18	22-1-18
**Station**	St-8	St-8	St-8	St-8	St-8	St-8	St-8	St-8	St-4	St-2	St-4	St-2	St-4
**∑MFlu**	9.2	1.7	<loq	<loq	<loq	24	<loq	24	2.0	10	0.44	4.0	0.56
**DBT**	6.0	4.5	0.97	1.8	<loq	1.2	4.5	17	<loq	<loq	<loq	<loq	<loq
**∑MDBT**	12	12	2.9	4.7	<loq	2.3	3.6	1.9	<loq	0.84	<loq	<loq	<loq
**Phe**	127	75	21	<loq	33	102	146	822	6.1	26	<loq	15	<loq
**∑MPhe**	25	27	10	25	26	35	27	182	13	48	7.1	22	6.3
**∑DMPhe**	17	26	15	41	39	29	23	42	19	69	10	26	6.9
**∑TMPhe**	5.9	11	7.3	9.3	24	12	9.3	8.0	11	45	30	8.7	1.6
**Ant**	10	6.4	<loq	3.5	3.2	4.3	6.2	21	0.36	2.8	<loq	0.66	<loq
**Flt**	151	56	<loq	30	29	61	21	622	4.3	25	2.8	7.0	3.2
**Pyr**	91	16	6.7	21	21	59	16	227	4.1	46	4.2	11	4.2
**∑MPyr**	10	5.3	2.8	3.3	8.3	4.0	4.2	17	4.2	19	9.3	3.3	0.9
**∑DMPyr**	3.2	5.5	2.0	4.9	15	4.5	8.1	4.3	3.8	30	10	3.8	0.2
**B[g,h,i]f**	5.4	<loq	<loq	<loq	<loq	<loq	<loq	<loq	0.89	20	<loq	<loq	<loq
**B[a]ant**	34	<loq	<loq	<loq	<loq	<loq	<loq	<loq	1.8	4.7	<loq	n.q.	n.q.
**Cry**	34	<loq	<loq	<loq	11	6.2	4.8	11	3.5	17	12	4.5	1.6
**∑MCry**	3.6	<loq	<loq	<loq	7.7	<loq	4.1	<loq	5.3	30	14	5.5	1.2
**B[b]f**	16	1.0	4.0	0.7	1.8	2.1	1.0	2.4	0.8	7.2	3.0	1.5	0.4
**B[k]f**	18	n.q.	n.q.	3.6	1.5	1.6	0.75	1.5	0.29	2.3	2.3	0.39	0.23
**B[e]pyr**	19	n.q.	n.q.	<loq	<loq	<loq	<loq	<loq	2.0	16	4.7	2.4	0.51
**B[a]pyr**	14	n.q.	n.q.	n.q.	n.q.	n.q.	n.q.	<loq	0.59	7.0	2.0	0.72	0.33
**Pery**	6.3	<loq	<loq	<loq	<loq	<loq	<loq	<loq	0.69	1.4	<loq	0.85	0.34
**In[1,2,3-cd]pyr**	12	<loq	<loq	<loq	<loq	<loq	n.q.	<loq	0.90	10	3.1	1.1	0.47
**Dib[a,h]ant**	<loq	n.q.	n.q.	n.q.	n.q.	n.q.	n.q.	n.q.	0.16	0.84	0.30	0.14	0.03
**B[g,h,i]pery**	13	<loq	<loq	<loq	<loq	<loq	<loq	<loq	1.4	39	5.3	2.1	1.2

Date	23-1-18	23-1-18	24-1-18	27-1-18	29-1-18	3-2-18	5-2-18	8-2-18	16-2-18	23-2-18	28-2-18
**Station**	St-6	St-7	St-2	St-2	St-4	St-2	St-4	St-1	St-4	St-4	St-5
**∑MFlu**	3.2	0.30	3.1	2.8	0.48	1.9	0.66	3.4	0.9	1.0	1.2
**DBT**	<loq	<loq	<loq	<loq	<loq	<loq	<loq	<loq	<loq	<loq	<loq
**∑MDBT**	<loq	<loq	<loq	<loq	<loq	<loq	<loq	<loq	<loq	<loq	<loq
**Phe**	11	3.4	17	12	<loq	5.5	<loq	14	6.9	7.0	5.3
**∑MPhe**	15	2.9	19	15	8.1	9.5	8.9	18	6.5	12	8.1
**∑DMPhe**	22	3.0	25	18	10	9.2	12	27	6.5	16	9.3
**∑TMPhe**	12	1.4	11	7.3	5.3	5.1	6.5	22	3.3	20	5.3
**Ant**	0.75	<loq	0.76	1.4	0.86	0.32	0.76	1.1	0.49	0.72	0.28
**Flt**	4.2	1.0	6.3	3.4	2.3	2.0	3.3	10	5.1	5.1	5.5
**Pyr**	6.4	1.7	8.5	4.5	3.0	1.8	5.5	11	5.4	4.5	7.1
**∑MPyr**	5.8	0.9	4.8	3.2	2.5	2.5	2.8	8.1	2.0	9.5	2.6
**∑DMPyr**	4.8	0.5	3.0	2.6	1.6	1.7	1.5	6.6	0.9	6.0	1.5
**B[g,h,i]f**	<loq	<loq	<loq	<loq	<loq	<loq	<loq	3.3	<loq	<loq	<loq
**B[a]ant**	2.3	n.q.	<loq	<loq	<loq	<loq	n.q.	7.9	n.q.	<loq	<loq
**Cry**	4.3	<loq	5.7	3.0	<loq	1.8	<loq	13	<loq	5.8	2.8
**∑MCry**	3.2	<loq	2.7	<loq	<loq	1.1	<loq	10	<loq	<loq	<loq
**B[b]f**	<loq	<loq	1.8	<loq	<loq	<loq	<loq	5.5	<loq	<loq	<loq
**B[k]f**	0.59	0.18	0.56	0.66	0.44	0.17	0.82	3.9	0.19	1.4	0.32
**B[e]pyr**	2.6	<loq	2.1	<loq	<loq	<loq	<loq	10	<loq	<loq	<loq
**B[a]pyr**	<loq	n.q.	<loq	<loq	<loq	<loq	<loq	4.2	<loq	<loq	<loq
**Pery**	<loq	<loq	<loq	<loq	<loq	<loq	<loq	2.0	<loq	<loq	<loq
**In[1,2,3-cd]pyr**	<loq	<loq	<loq	<loq	<loq	<loq	<loq	3.6	<loq	<loq	<loq
**Dib[a,h]ant**	<loq	n.q.	<loq	<loq	n.q.	<loq	<loq	<loq	n.q.	<loq	n.q.
**B[g,h,i]pery**	4.4	<loq	2.2	<loq	<loq	<loq	<loq	5.0	<loq	<loq	<loq

Table S8: Classification of the PAHs analyzed in this study as low molecular weight (LMW) PAH or high molecular weight (HMW) PAH according to the number of aromatic rings and molecular weight.

LMW	HMW
∑MFlu	B[g,h,i]f
DBT	B[a]ant
∑MDBT	Cry
Phe	∑MCry
∑MPhe	B[b]f
∑DMPhe	B[k]f
∑TMPhe	B[e]pyr
Ant	B[a]pyr
Flt	Pery
Pyr	In[1,2,3-cd]pyr
∑MPyr	Dib[a,h]ant
∑DMPyr	B[g,h,i]pery

Table S9: Relative abundances (%) of HCB taxa, expressed as the mean, standard deviation, maximum and minimum of each taxa for each campaign.

	Livingston 2015				Deception 2017				Livingston 2018			
**HCB**	**Mean**	**Sd**	**Max.**	**Min.**	**Mean**	**Sd**	**Max.**	**Min.**	**Mean**	**Sd**	**Max.**	**Min.**
HCB_Sulfitobacter	3.78	5.17	17.40	0.01	3.20	2.97	11.60	0.02	4.23	6.15	29.61	0.03
HCB_Psychrobacter	0.06	0.06	0.20	0.02	0.03	0.00	0.03	0.03	4.20	14.28	58.07	0.02
HCB_Bacillus									0.49	0.83	1.95	0.03
HCB_Pseudoalteromonas	1.03	1.09	3.49	0.02	0.02	0.01	0.03	0.02	0.24	0.41	1.64	0.02
HCB_Marinomonas	0.07	0.06	0.11	0.03					0.12	0.22	0.52	0.02
HCB_Flavobacterium	0.09	0.06	0.21	0.02	0.07	0.08	0.20	0.02	0.12	0.14	0.82	0.02
HCB_Pseudomonas	0.06	0.04	0.11	0.03	0.09	0.08	0.18	0.02	0.11	0.21	1.13	0.02
HCB_Rhodoferax	0.15		0.15	0.15					0.10	0.07	0.26	0.03
HCB_Nocardioides	0.02		0.02	0.02					0.09	0.16	0.46	0.02
HCB_Sphingobium									0.07	0.04	0.15	0.02
HCB_Colwellia	0.22	0.82	7.58	0.02	0.05	0.02	0.08	0.04	0.07	0.13	0.90	0.02
HCB_Alkanindiges	0.07		0.07	0.07	0.12	0.05	0.16	0.07	0.07	0.05	0.17	0.02
HCB_Shewanella	0.13	0.19	0.70	0.02	0.07		0.07	0.07	0.07	0.05	0.14	0.02
HCB_Jannaschia									0.07	0.09	0.17	0.02
HCB_Arcobacter	0.22	0.13	0.31	0.13					0.07		0.07	0.07
HCB_Sulfuricurvum	0.15	0.09	0.21	0.05					0.05	0.04	0.13	0.02
HCB_Sphingomonas									0.05	0.06	0.21	0.02
HCB_Arenimonas									0.05	0.00	0.05	0.05
HCB_Polaromonas	0.07	0.03	0.11	0.02					0.05	0.02	0.08	0.02
HCB_Novosphingobium									0.05	0.03	0.08	0.02
HCB_Arthrobacter									0.05	0.04	0.08	0.02
HCB_Thiobacillus	0.05	0.03	0.08	0.02					0.04	0.02	0.07	0.02
HCB_Rhodococcus									0.04	0.02	0.08	0.02
HCB_Woeseia	0.06		0.06	0.06					0.04	0.02	0.05	0.02
HCB_Ralstonia									0.03		0.03	0.03
HCB_Glaciecola	0.03		0.03	0.03					0.03	0.00	0.03	0.03
HCB_Psychrilyobacter	0.03	0.02	0.06	0.02					0.03	0.02	0.05	0.02
HCB_Bacteroides	0.04	0.02	0.07	0.02					0.03	0.02	0.08	0.02
HCB_Thalassotalea	0.04	0.03	0.08	0.02					0.03	0.01	0.05	0.02
HCB_Acinetobacter	0.25	0.05	0.29	0.20					0.03	0.01	0.05	0.02
HCB_Nonlabens	0.09	0.03	0.11	0.07					0.03	0.02	0.05	0.02
HCB_Faecalibacterium									0.03	0.01	0.05	0.02
HCB_Paludibacter	0.02		0.02	0.02					0.02	0.01	0.03	0.02
HCB_Lachnoclostridium									0.02		0.02	0.02
HCB_Proteiniphilum									0.02		0.02	0.02
HCB_Desulfobacterium	0.02		0.02	0.02					0.02		0.02	0.02

HCB_Bdellovibrio									0.02	0.00	0.02	0.02
HCB_Geobacter									0.02		0.02	0.02
HCB_Cutibacterium	0.14		0.14	0.14								
HCB_Vibrio	0.10	0.10	0.21	0.03								
HCB_Oleispira	0.10	0.05	0.13	0.07								
HCB_Halomonas	0.03		0.03	0.03								
HCB_Cytophaga	0.02		0.02	0.02								
HCB_Marinobacter	0.02		0.02	0.02								
HCB_Thauera	0.02		0.02	0.02								

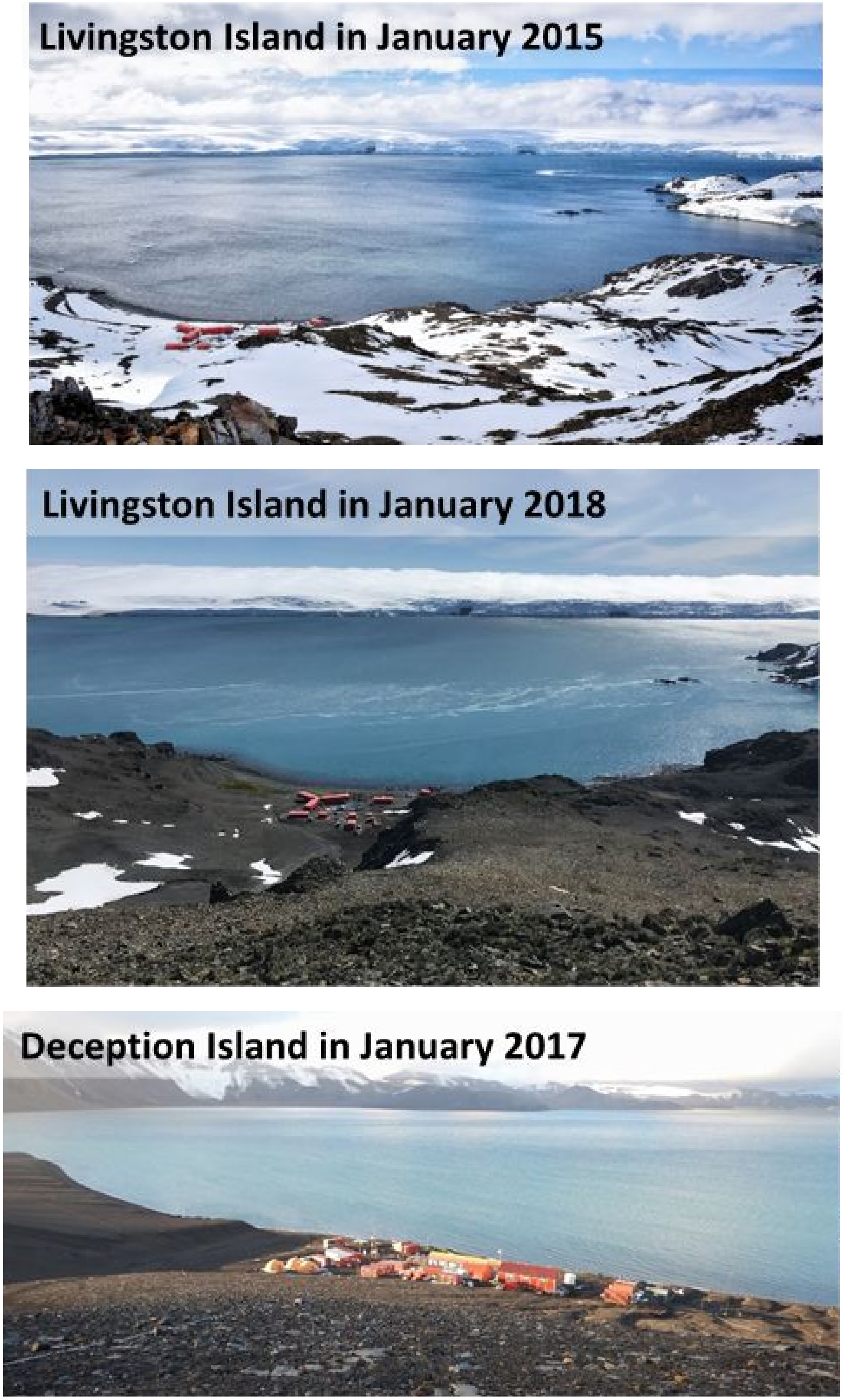

Figure S1: Picture of South bay for the 2015, 2017 and 2018 campaigns (January) showing the difference in snow cover.

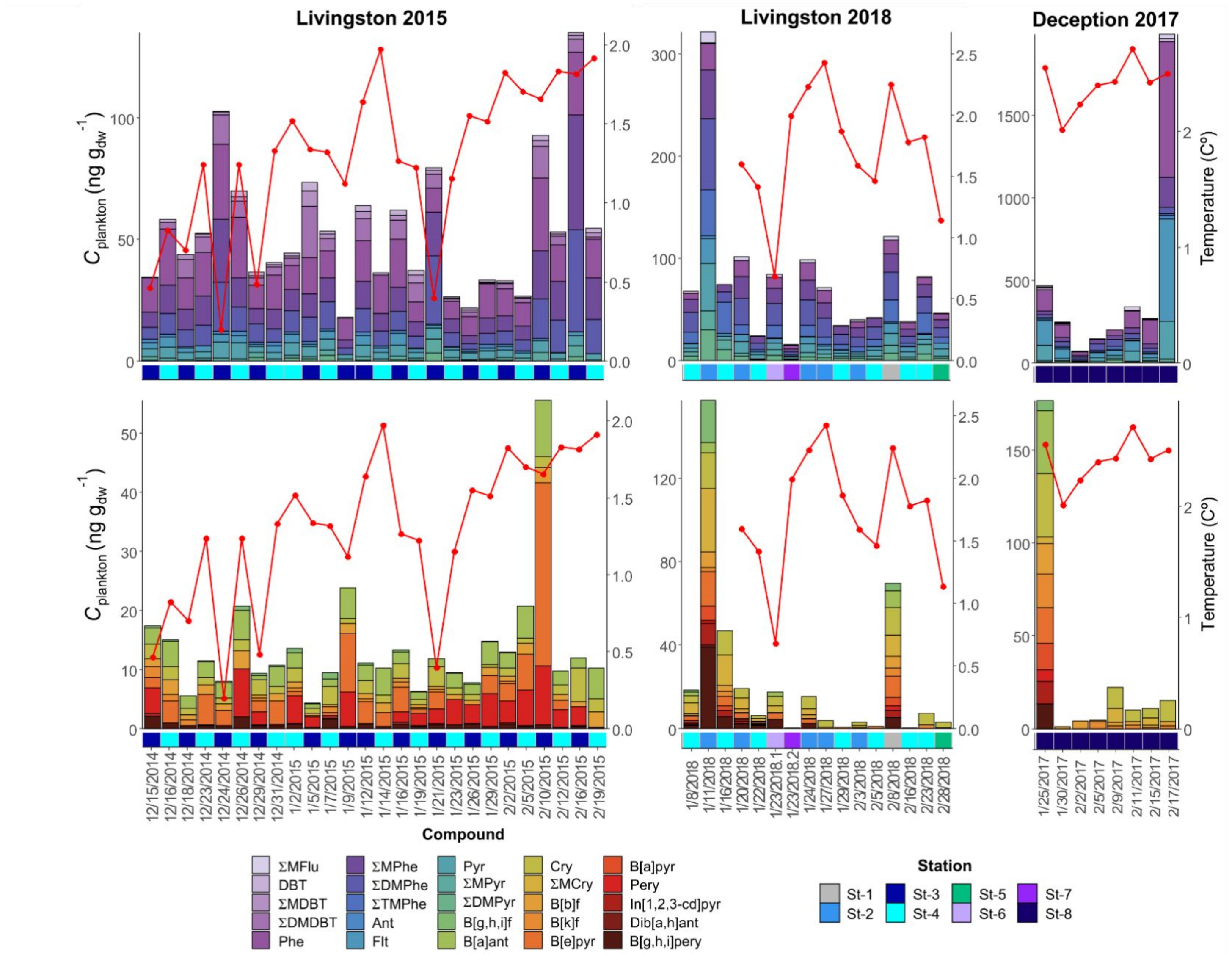

Figure S2: Occurrence of individual low MW PAHs (upper panels) and high MW PAHs (lower panels) in the plankton phase for the three sampling campaigns. Red line indicates seawater temperature.

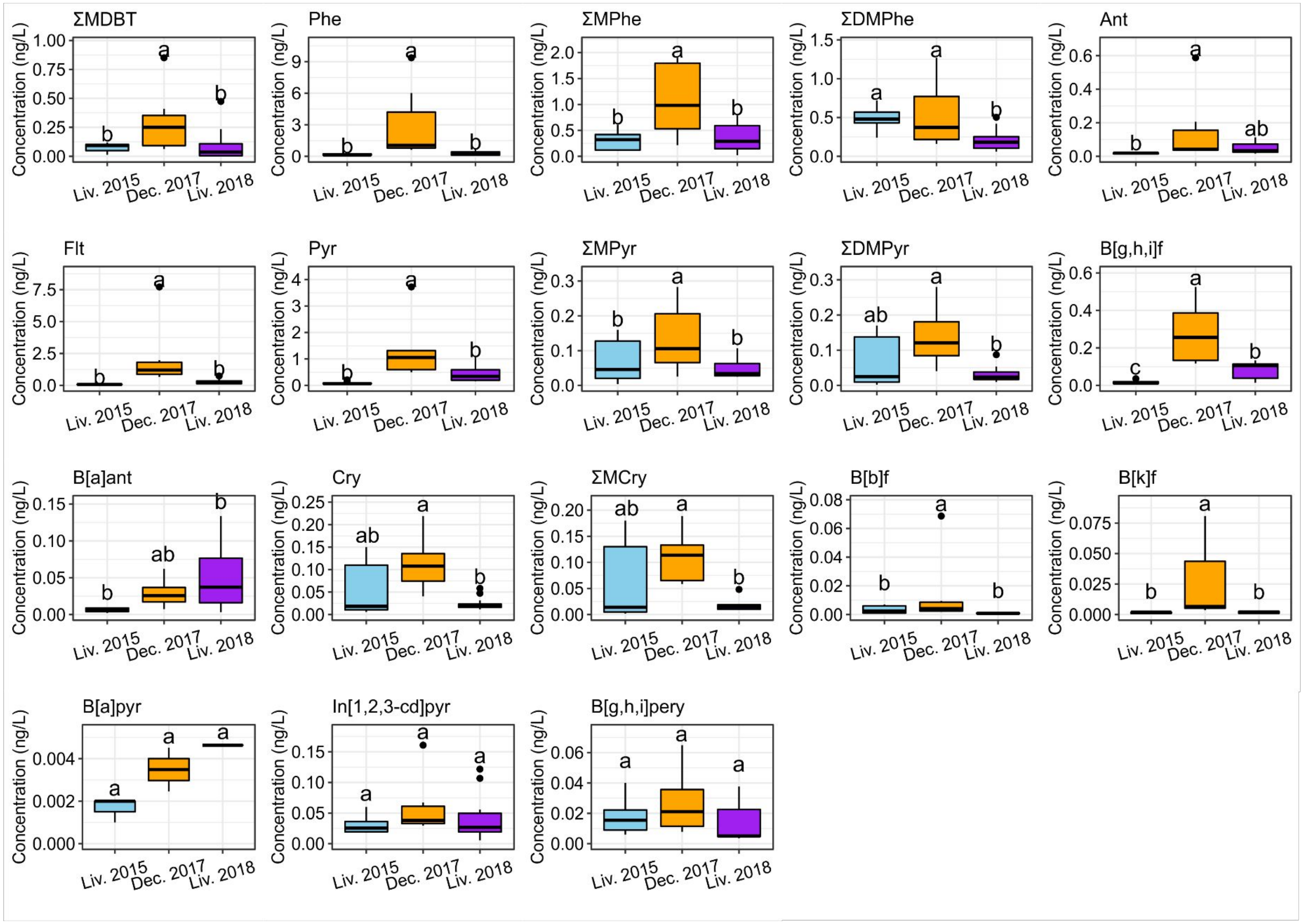

Figure S3: Differences in individual PAH concentrations in the water dissolved phase for the three sampling campaigns assessed by an ANOVA followed by a Tukey test. Significant differences ($p < 0.05$) are indicated with different letters.

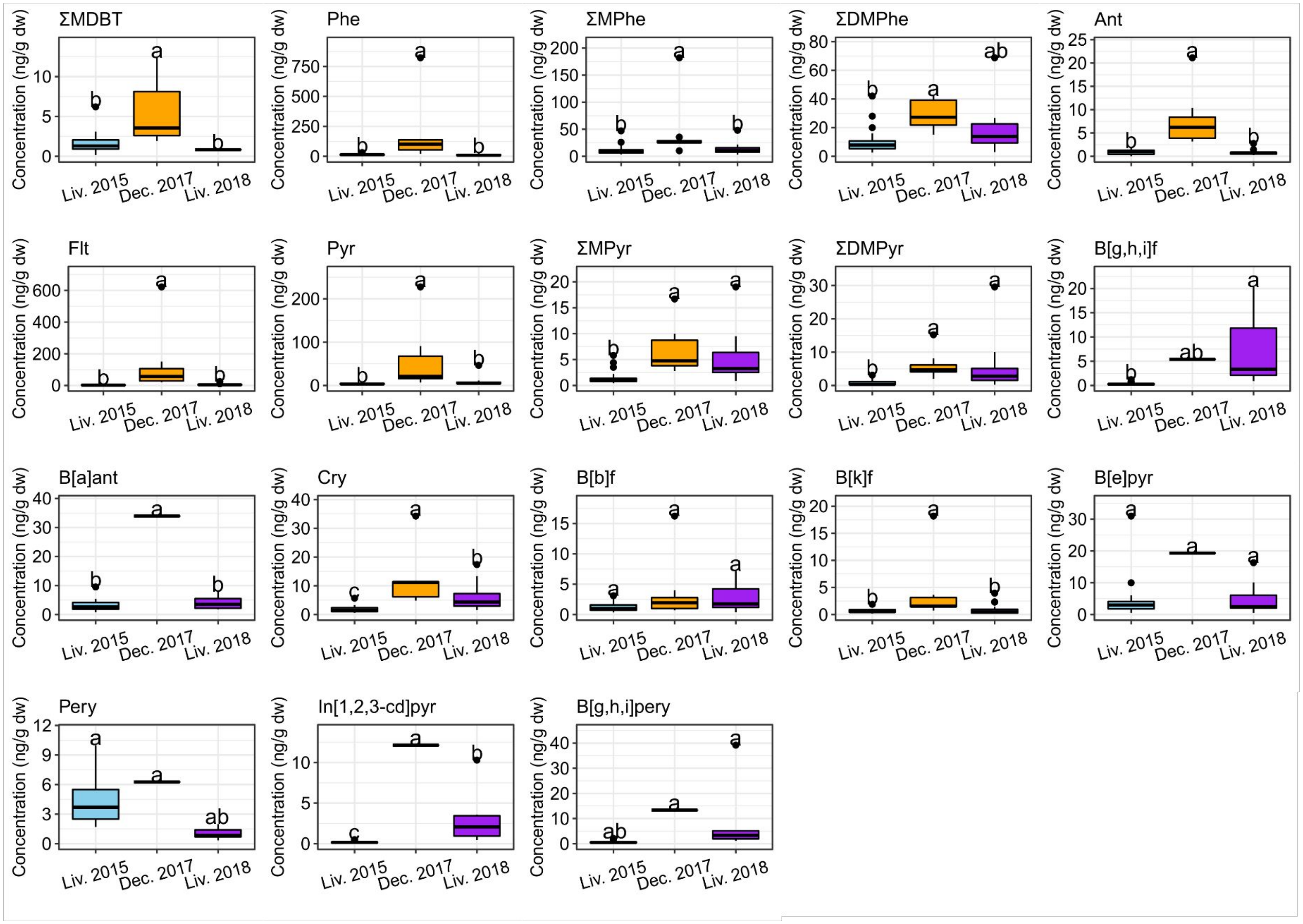

Figure S4: Differences in individual PAH concentrations in the plankton phase for the three sampling campaigns assessed by an ANOVA followed by a Tukey test. Significant differences ($p < 0.05$) are indicated with different letters.

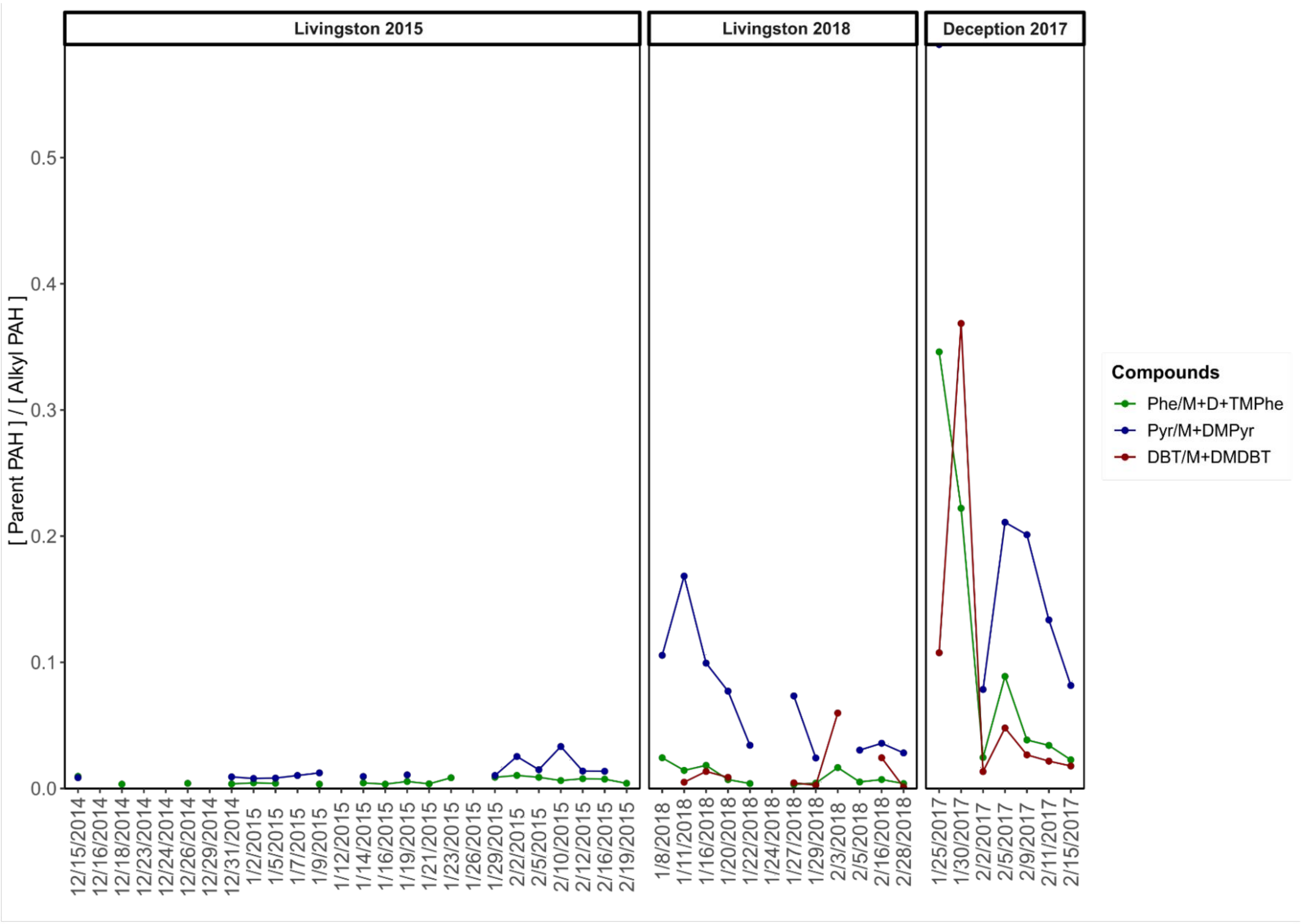

Figure S5: Temporal series of the parent/alkyl-PAHs concentration ratios in the water dissolved phase for the three campaigns. The ratios for the phenanthrene, pyrene and dibenzothiophene and their methylated forms are shown in green, blue and red, respectively.

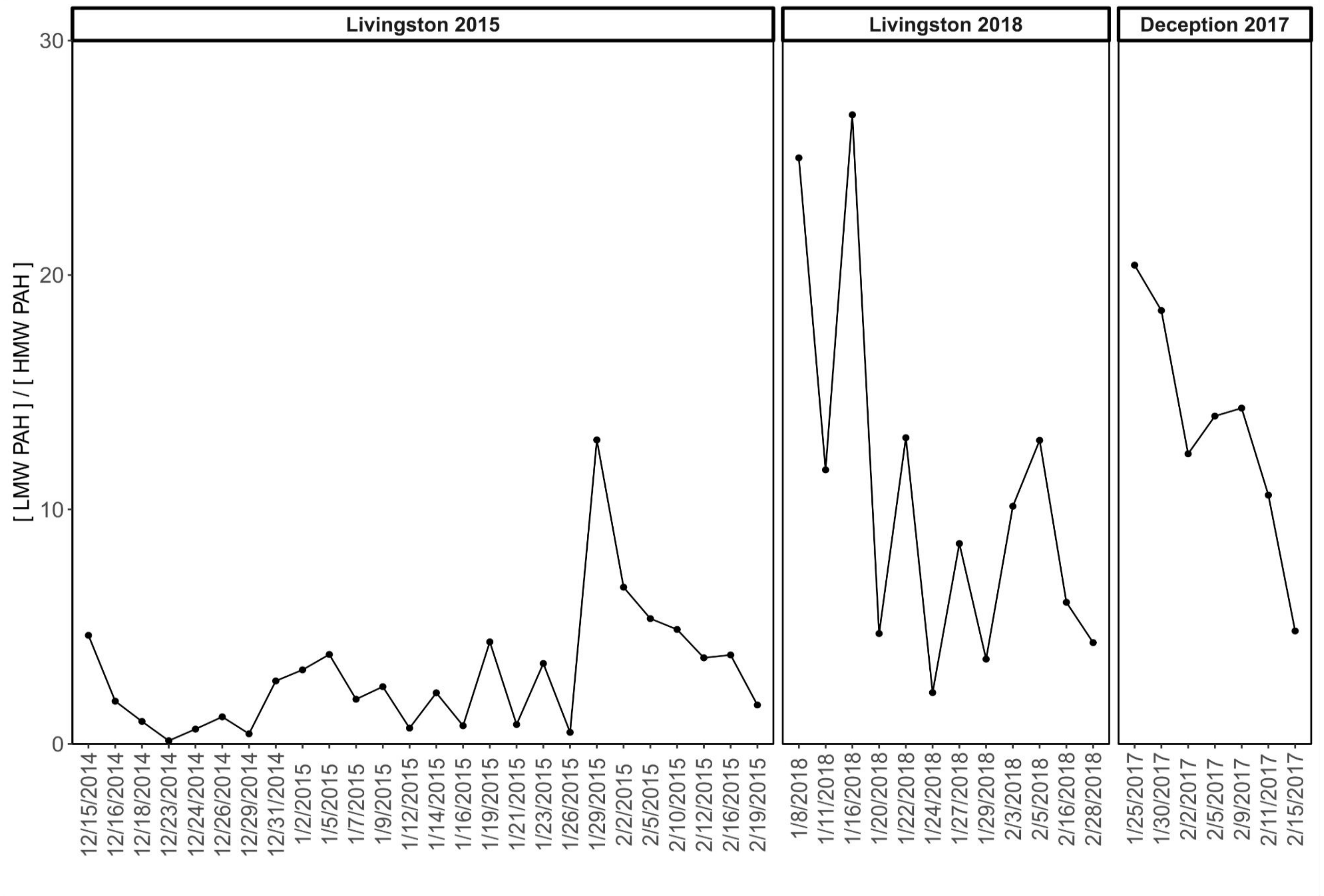

Figure S6: Temporal series of the LMW/HMW ratios in the water dissolved phase for the three campaigns.

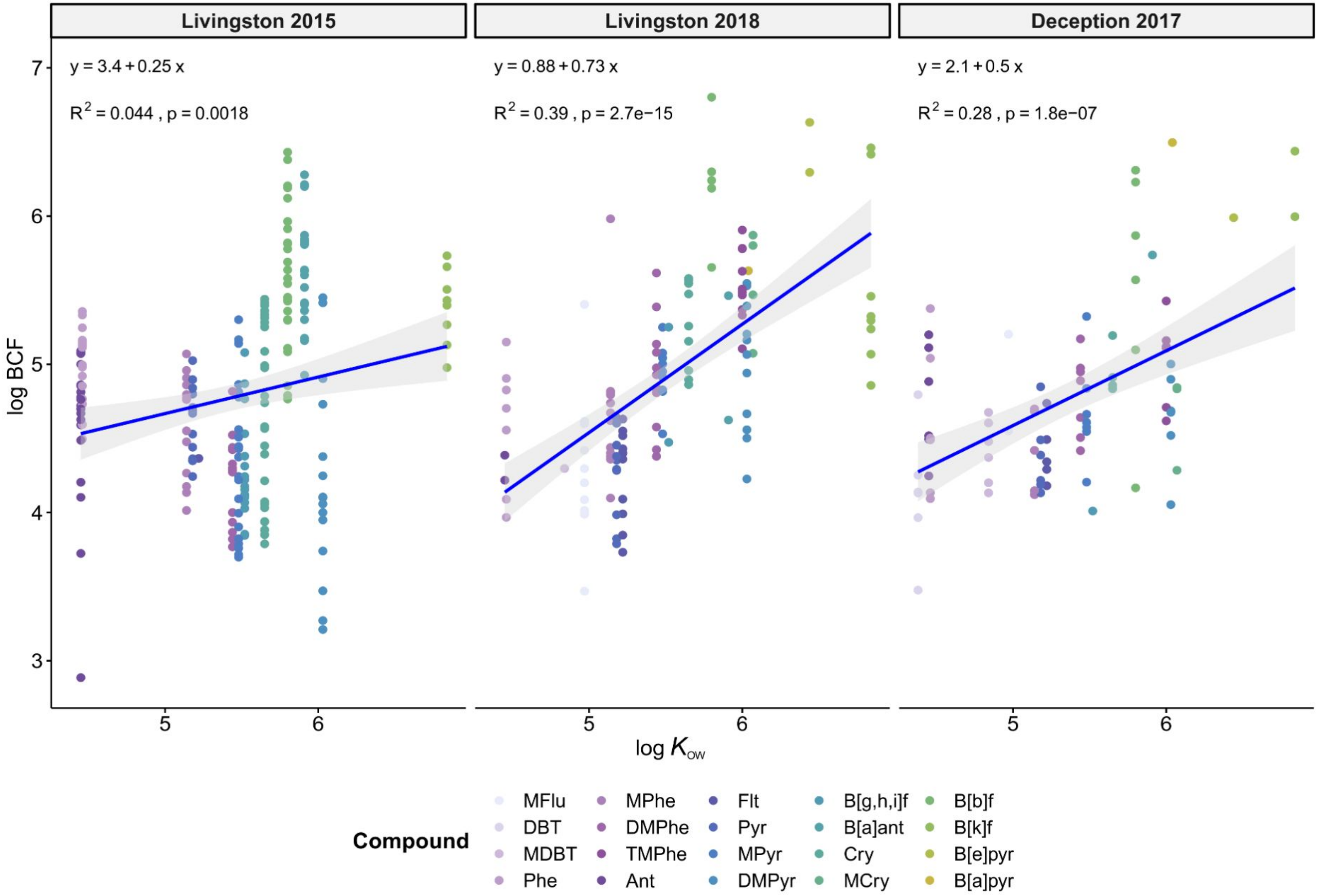

Figure S7: Bioconcentration factors (Log BCF) versus octanol-water partition coefficients (Log K_{ow}) for the three sampling campaigns.

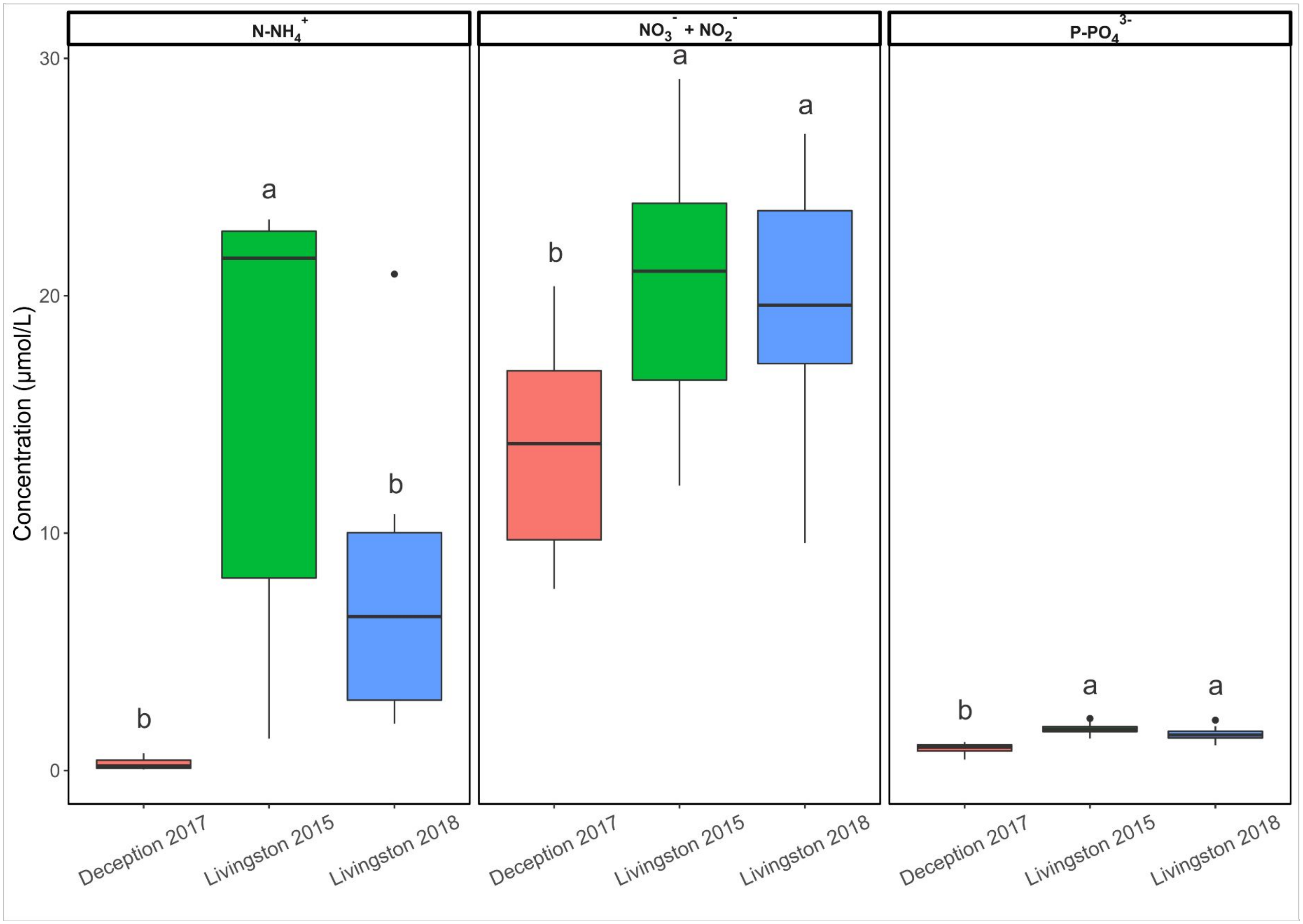

Figure S8: Boxplot of inorganic nutrient concentrations for the three campaigns. Letters indicate significant differences ($p < 0.05$) obtained with an ANOVA test followed by a Tukey test.

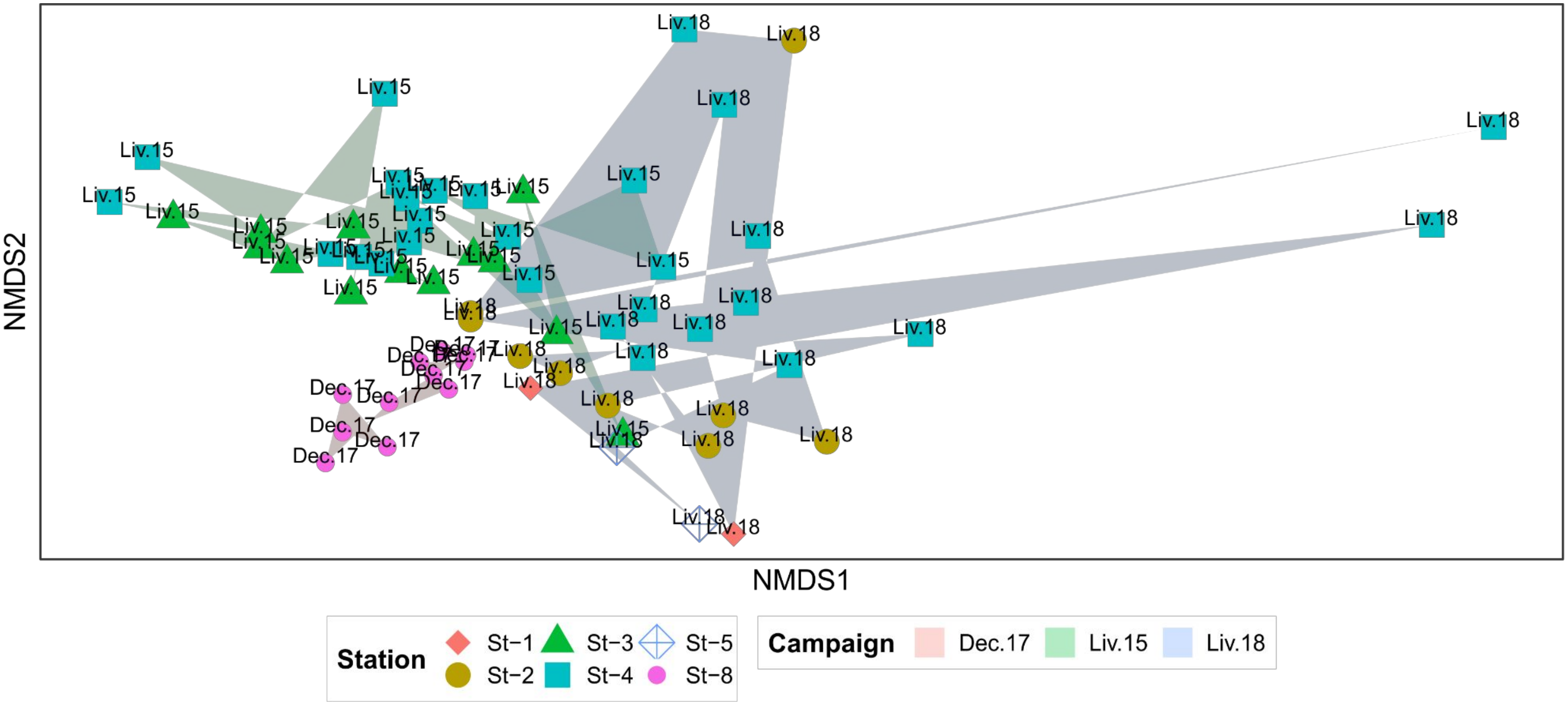

Figure S9: Non-metric Multidimensional Scaling (NMDS) plot for clustering samples according to their ASV composition. Background colors represent each of the sampling campaigns (Livingston 2015 in green, Livingston 2018 in blue and Deception 2017 in red), while colored shapes represent different sampling stations.

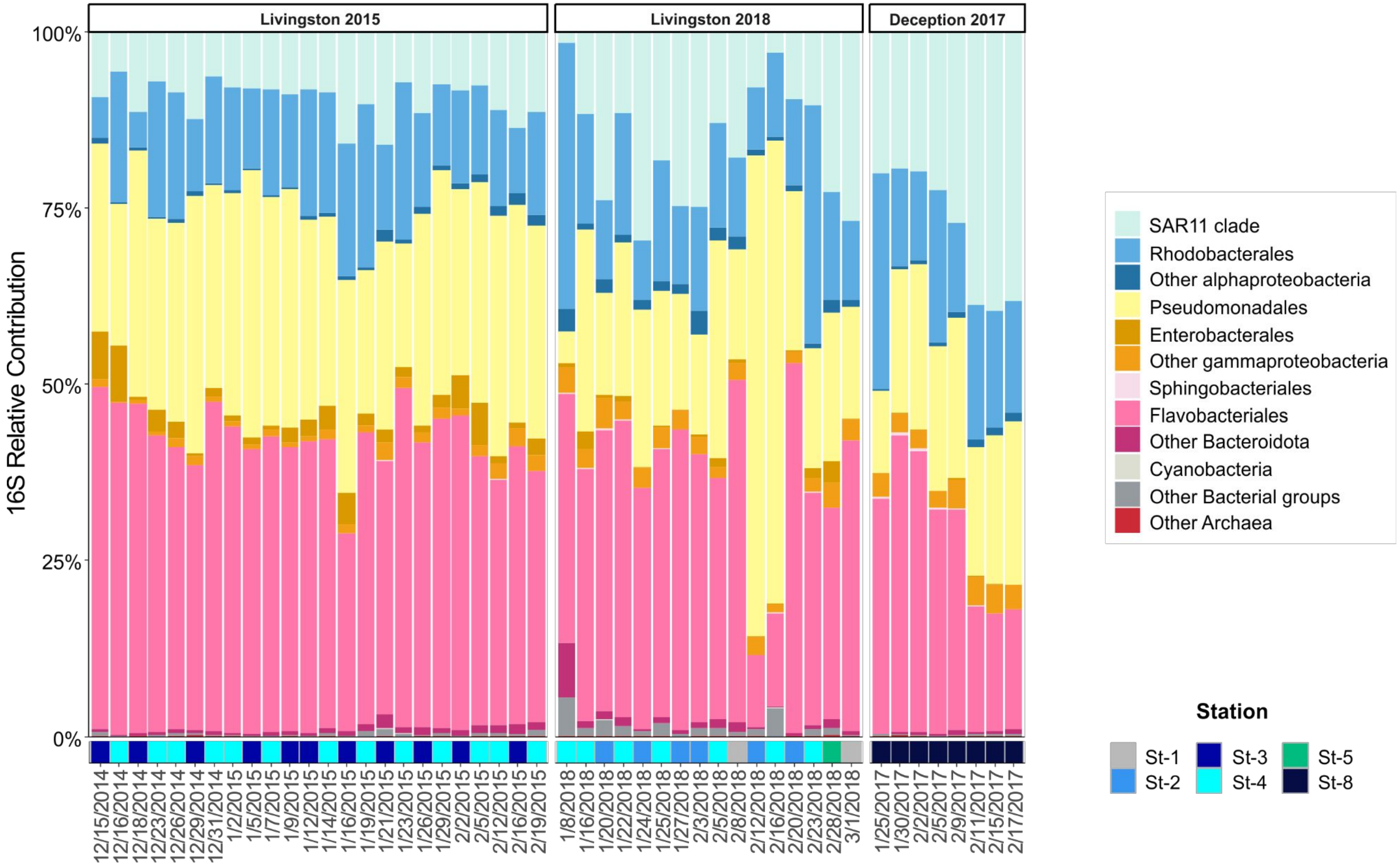

Figure S10: Structure of the bacterial community for the three sampling campaigns.

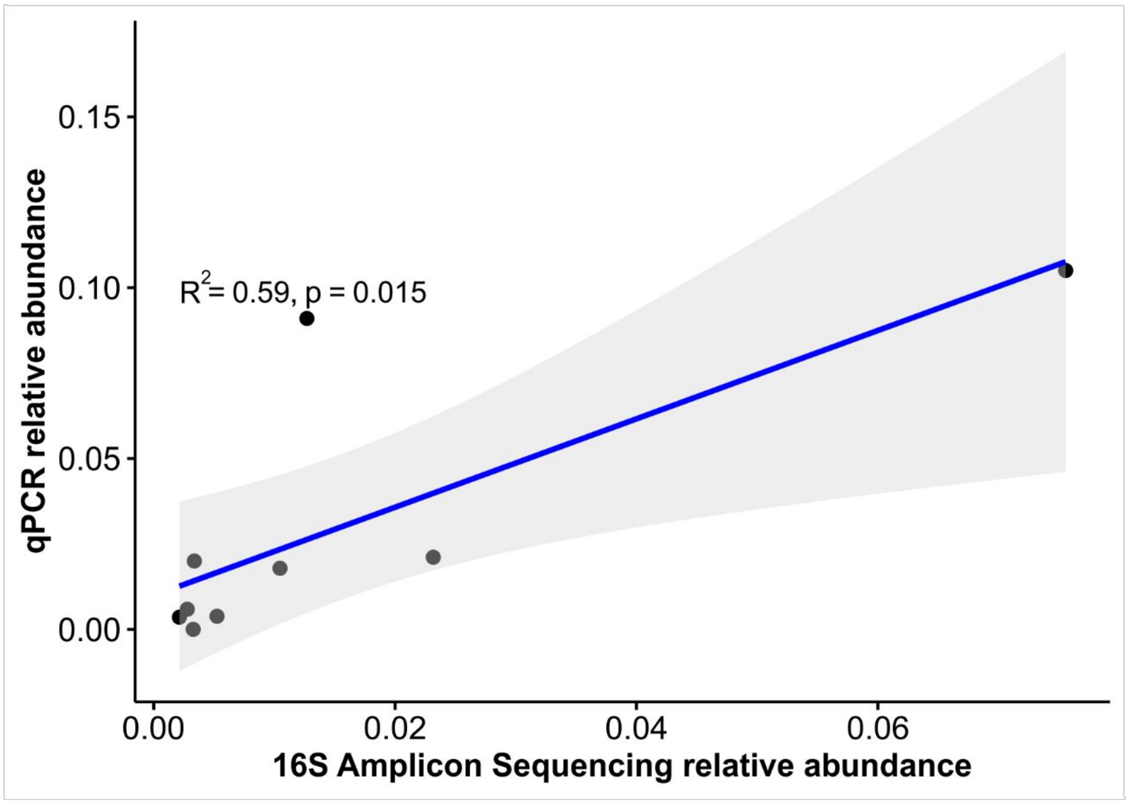

Figure S11: Correlation between amplicon sequencing relative abundance and qPCR relative abundance for the genus *Colwellia* at Jhonsons dock.

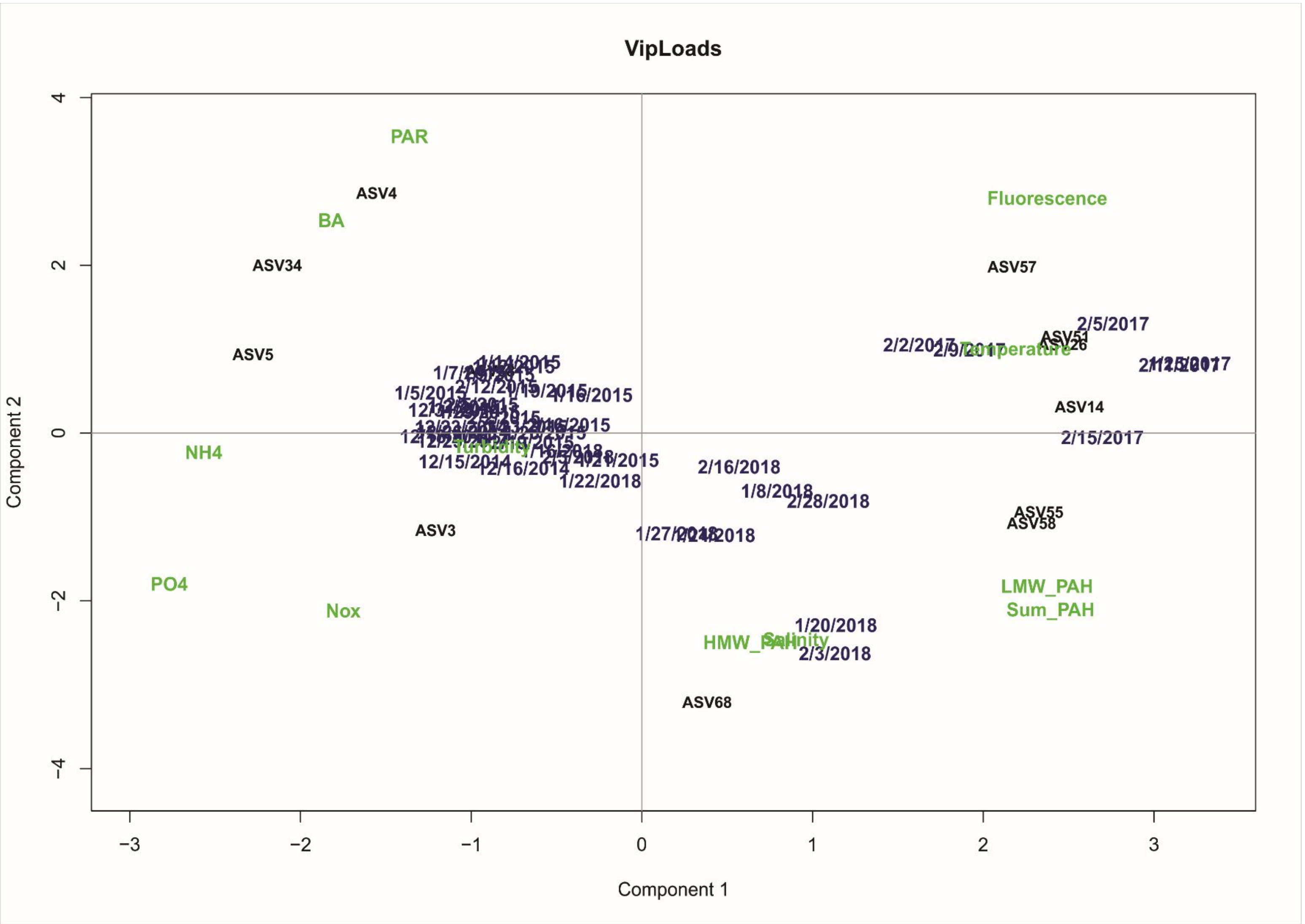

Figure S12: Partial least squares regression (PLS): In black: VIP X loadings from a matrix of ASV abundancies. In blue: x scores plot showing the distribution of water samples according to the bacterial community composition. In green: VIP y loadings of PAH concentrations and other environmental variables.

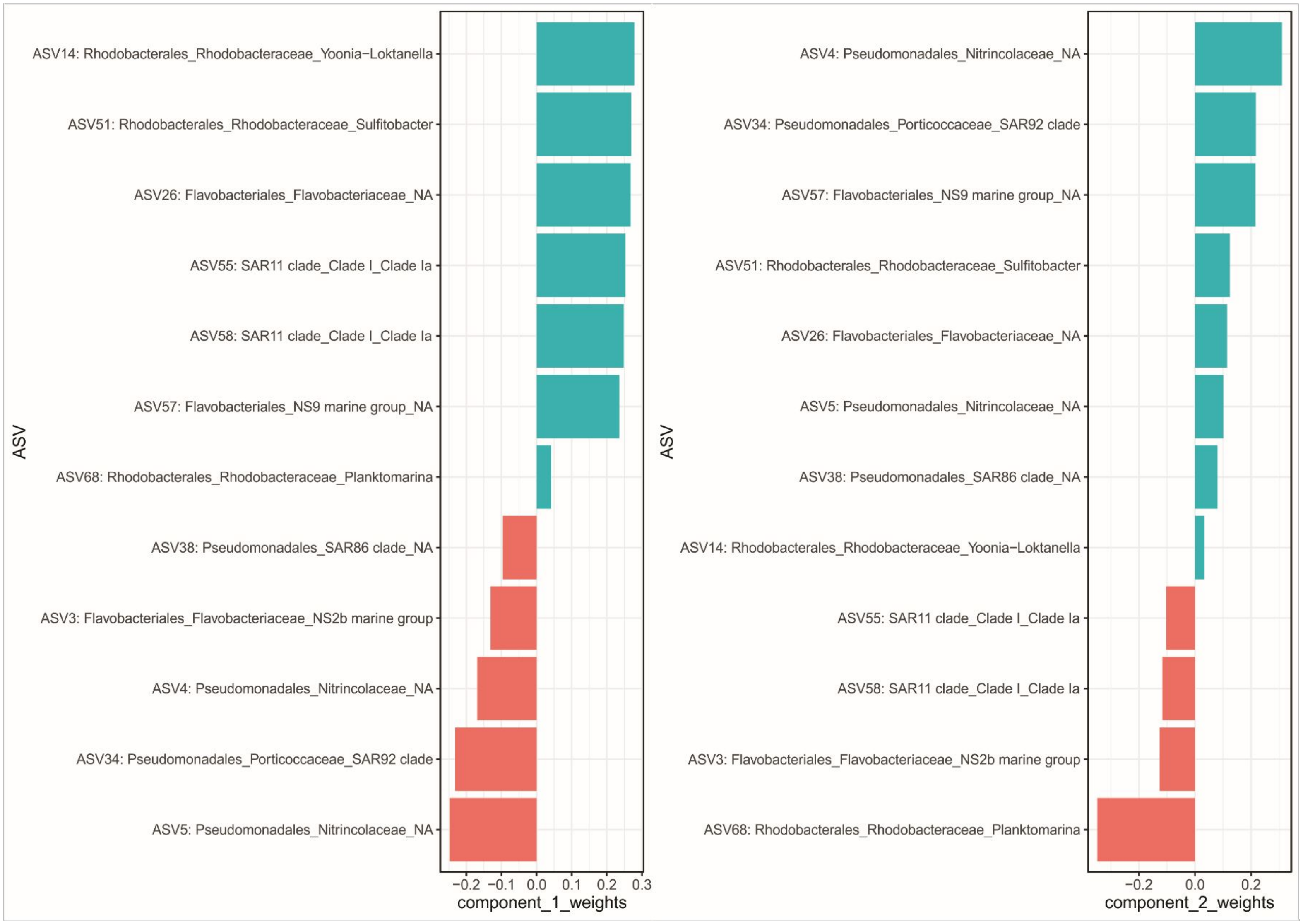

Figure S13: Plot showing the weight of the VIP x variables (individual ASV) over the components 1 and 2 of the PLS.

References

(1) Casal, P.; Cabrerizo, A.; Vila-Costa, M.; Pizarro, M.; Jiménez, B.; Dachs, J. Pivotal Role of Snow Deposition and Melting Driving Fluxes of Polycyclic Aromatic Hydrocarbons at Coastal Livingston Island (Antarctica). *Environmental Science & Technology* **2018**, *52* (21), 12327–12337.
